# Synthesis, characterization, and *in silico* and *in vivo* profiling of selective cyclo-oxygenase-2 inhibitors of indazole–indolinone derivatives with anti-inflammatory and analgesic potency

**DOI:** 10.3389/fphar.2025.1723200

**Published:** 2026-01-30

**Authors:** Sultan Ibrahim Alkubaysi, Mohammad Jaffar Sadiq Mantargi, Faisal Ateeq Almalki, Alaa Mohammad Alqahtani, Alaa Omar Baryyan, Saeed Mohammed Tayeb, Hussni Ahmad Muathen

**Affiliations:** 1 Department of Pharmaceutical Sciences, College of Pharmacy, Umm Al-Qura University, Makkah, Saudi Arabia; 2 Department of Pharmaceutical Sciences, Pharmacy Program, Batterjee Medical College, Jeddah, Saudi Arabia

**Keywords:** analgesic, anti-inflammatory, drug development, *in silico*, molecular docking, molecular dynamic simulations, preclinical study

## Abstract

**Background:**

The intensive use of non-steroidal anti-inflammatory and analgesic drugs (NSAIDS) worldwide poses a challenge to scientists because of the adverse side effects. This article aims to synthesize a novel group of 1-aminoindazole-isatin Schiff base compounds considering their potency as analgesic and anti-inflammatory agents.

**Methods:**

The synthesis of novel agents involved reflux condensation of isatin derivatives (5 mmol) and 1-aminoindazole (5 mmol) in ethanol for 2 h, which were then characterized for their structural integrity. *In silico* evaluation using PyRx, BIOVIA Discovery Studio, and GROMACS was performed to determine the affinity of the specific receptors and compare them with the results gained by use of standard diclofenac before preclinical evaluation using albino mice (analgesic activity) and rats (anti-inflammatory activity). The preclinical analgesic potency was analyzed via Eddy’s hot plate and tail-pin methods, whereas, the anti-inflammatory potency was analyzed through carrageenan-induced paw edema against diclofenac as the standard agent.

**Results:**

A high percentage yield of the reactions was determined (≈80%); the IR, NMR, and mass spectra showed the compounds to be stable with no shifts, justifying the accuracy of the procedure employed. The molecular docking of the ligands with two different crystal structures of proteins of interest, i.e., COX-1 and COX-2, yielded stable and the lowest binding energies, i.e., −9.6 kcal/mol for AB 12 and – 7.1 kcal/mol for diclofenac. Through molecular dynamic simulations employing GROMACS for a time period of 50 ns, AB 12 and diclofenac also yielded a thermodynamically stable and structurally folded protein and ligand complex, showing an average of 0–3 (AB 12) and 0–5 (diclofenac) hydrogen bonds with the least system fluctuations and atom deviations; furthermore, the potential energy of the complete system was stabilized at an average point of – 685,000 kj/mol for both molecules. The preclinical results showed a significant value for the ligand AB 12 (p ≤ 0.01) against the diseased control group.

**Discussion:**

The ligand AB 12, AB 14 and AB 15 is exceptional as an analgesic and anti-inflammatory agent. AB 12 further showed stable hydrogen bonds with protein COX-2 for 50 ns in comparison with diclofenac. Based on this study, these molecules can be considered best for future studies regarding the toxicological profile.

## 1 Introduction

Non-steroidal anti-inflammatory drugs (NSAIDS) are a group of medications that are widely used in treating pain, inflammation, and fever. They are usually classified into two groups depending on their structure and mechanism of action (Bacchi et al., 2012; Vane and Botting, 1998): non-selective NSAIDS, derived from propionic acid, acetic acid, and anthranilic acid, such as ibuprofen (1), diclofenac (2), and mefenamic acid (3), respectively. They inhibit the activities of both cyclooxygenase enzymes (COX-1 and COX-2) present in the body (Ghlichloo and Gerriets, 2023).

The second group, selective NSAIDS, inhibits the activity of only the COX-2 enzyme and has a milder effect on the gastrointestinal system. It includes celecoxib (4), parecoxib (5), and meloxicam (6). COX-1 is a natural variant that produces prostaglandins that are essential for keeping the body in homeostasis, especially for maintaining gastric mucosa health, platelet aggregation, and kidney function. Conversely, COX-2 is mostly an inducible variant that is turned on by pro-inflammatory signals at sites of inflammation. It is also expressed in specific tissues. These differences in regulation and function, along with differences in inhibitor selectivity, help explain why inhibition of selective COX-2 has a better therapeutic profile and fewer gastrointestinal side effects than non-selective NSAIDs (Vane and Botting, 1998; Sharma et al., 2020).

During the last several years, many research works have been conducted on designing and synthesizing a series of novel compounds possessing analgesic and anti-inflammatory properties so that they will inspire scientists to develop new, more potent analgesic drugs with less adverse side effects (Kajal et al., 2014; Shaker et al., 2023).

Pyrazole and its benzo derivatives (indazoles) have high synthetic value as an intermediate in the preparation of some pharmacological drugs, such as the analgesic Celebrex and anti-inflammatory COX-2 celecoxib (4) and bendazac (7), for treating joint and muscle pain (Bekhit et al., 2021; Cheekavolu and Muniappan, 2016).

**FIGURE 1:**
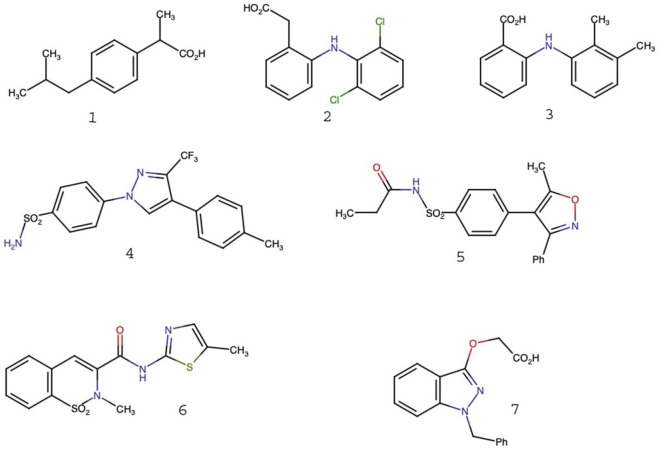
Represents the 2D structure of standard NSAIDS, i.e., ibuprofen (1), diclofenac (2), mefenamic acid (3), celecoxib (4), parecoxib (5), meloxicam (6), and bendazac (7).

1H-Indole-2,3-dione (isatin) (8) (Figure 1) is a privileged core of many bioactive heterocyclic agents (Mi̇shra et al., 2021). The therapeutic anti-inflammatory actions of isatin-based agents have drawn the interest of many scientists (Dantas et al., 2020). Their hydrazone derivatives have received considerable attention for their biological activities due to the presence of (-C=N) bonds (Singh, 2016; Aziz, 2020). Several isatin–hydrazone derivatives have shown anti-inflammatory properties (Zeeshan et al., 2019; Warnasih et al., 2025).

Owing to these findings, and since N-aminoazoles have demonstrated various chemical activities (Dyablo et al., 2002), we designed a structure that contains both active isatin and N-aminoindazole rings in a Schiff base form and study their biological anti-inflammatory and analgesic activities.

**FIGURE 2:**
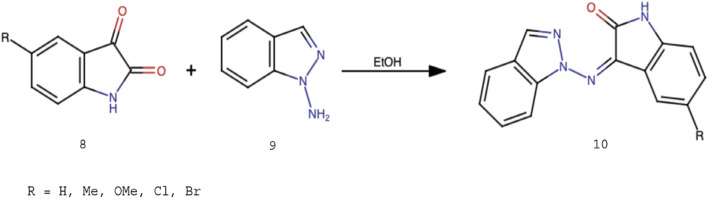
Represents the scheme of synthesis of the desired hydrazones (Scheme 1).

The target molecules can be synthesized by reflux condensation of an equimolar amount of isatin derivatives (8) and a 1-aminoindazole (9) in ethanol to produce the corresponding hydrazones (10) (Figure 2).

The selection of isatin derivatives was determined by the impact of their substituents on the activity. Electron-donating groups (such as methyl and methoxy) could improve lipophilicity and enhance pain relief, while electron-withdrawing groups (such as chloro and bromo) may enhance binding interactions in the COX-2 pocket. These variations facilitate the analysis of the structure–activity relationship (SAR) to identify derivatives exhibiting enhanced anti-inflammatory and analgesic properties.

Recent advancements in computational studies such as molecular docking help in predicting the possible potency of the molecule by identifying strong binding affinities, and further molecular dynamic (MD) simulations assisting in understanding the behavior of ligands and proteins in the virtual cellular environment would result in generation of concrete evidence toward developing novel agents with safer pharmacokinetic and pharmacodynamic profiles for *in vivo* evaluation by rational use of rodents. We carried out a synthesis of novel 3-(indazole-1-ylimino)-1,3-dihydroindol-2-one derivatives designed via a scheme for COX-2 selectivity. These compounds were evaluated through a combination of methods such as molecular docking, MD simulations, and *in vivo* studies documenting the analgesic and anti-inflammatory efficacy. By integrating various approaches, this study aims to establish the therapeutic potential of these derivatives and strengthen the SAR that could shape the future analgesic and anti-inflammatory drug development.

This research analyzes whether structurally modified derivatives of 3-(indazole-1-ylimino)-1,3-dihydroindol-2-one can exhibit selective COX-2 selective, which may or may not result in potent anti-inflammatory and analgesic activity. The idea was based on the hypothesis that the addition of 1-aminoindazole into the isatin derivatives would increase COX-2 selectivity with improved pharmacological outcomes. We aimed to perform synthesis and characterization of the isatin derivatives based on the rationale design scheme, evaluation of the obtained molecule’s binding affinities through *in silico* tools, and assessment of the anti-inflammatory and analgesic activity through *in vivo* models and thereby establishing their potential as a lead compound for selective COX-2 inhibition.

## 2 Materials and methods

Isatin derivatives and 1-aminoindazole were purchased from Combi Blocks Chemicals, San Diego, USA. Aminoindazole can also be prepared from indazole and hydroxylamine-O-sulfonic acid according to literature method of Ali et al. (2013). Melting points were measured by using the Stuart SMP3 apparatus, infrared (IR) spectra were recorded on the Shimadzu IRAffinity-1S FT IR, and nuclear magnetic resonance (NMR) spectra were recorded on a Bruker Avance III 500 MHz spectrometer, mass spectrometer, and CHN analyzer.

### 2.1 General procedure for the synthesis of isatin-aminoindazole hydrazones

A mixture of isatin derivative (5 mmol) and 1-aminoindazole (5 mmol) in ethanol (5 mL) was stirred by refluxing for 2 h. After cooling to room temperature, the resultant solid was filtered by suction and washed with a small amount of petroleum ether. Recrystallization from ethanol afforded the corresponding hydrazone. The percentage reaction yield of all synthetic compounds ranged between 79% and 84%. A summary of the characterization methods employed, such as melting point determination, IR, NMR, mass spectrometry, and elemental analysis, is provided in the next section, and further detailed data are presented in the *Results* section.

### 2.2 Characterization of the synthetic derivatives

The melting points of the novel synthetic agents were measured with the Stuart SMP3 apparatus, and the results are reported without corrections. The infrared spectrum (IR report) was obtained through the “Shimadzu IRAffinity-1S FTIR spectrometer” using potassium bromide pellets. The NMR spectra were obtained using a “Bruker Avance III 500 MHz spectrometer,” with parts per million being considered for reporting the chemical shift relative to use of tetramethylsilane (TMS) as the internal standard. Mass spectra were obtained using the Shimadzu LCMS 8050 UFMS mass spectrophotometer, and elemental analysis was performed using a CHN analyzer to confirm the composition and purity of the synthesized compounds.

### 2.3 Molecular docking and molecular dynamic simulation

#### 2.3.1 Preparing the protein for *in silico* processing

By targeting the proteins of interest that play a significant role in regulating inflammation and pain, we aimed to determine the binding affinity of the best molecule out of five as a candidate for anti-inflammatory and analgesic potential for novel drug discovery and development in management of inflammation and pain; several proteins of interest were identified: for example, cyclo-oxygenase I (PDB ID: 6Y3C) and cyclo-oxygenase II (PDB ID: 5KIR), which were responsible for the development of chemical mediators of inflammation such as cytokines, leukotrienes, and thromboxane (Miciaccia et al., 2021; Orlando and Malkowski, 2016). The protein pdb files were downloaded from the RCSB PDB database (https://www.rcsb.org/) (Zardecki et al., 2021) and visualized through the BIOVIA Discovery Studios visualizer v.21.1.0.20298 (Alzarea et al., 2025) application, which helped in preparing the protein for molecular docking, and MD simulation by removing the HETATMs, water, co-crystals, and protein protonation was carried out through the introduction of polar hydrogens to the protein 3D structure to induce charge around the protein structure to facilitate perfect interaction with the ligand (Alzarea et al., 2025).

#### 2.3.2 Preparing ligands for molecular docking

The ligands were designed using ChemDraw 2D, and the molecules energies were stabilized, chirality was identified, and energy was minimized using ChemDraw 3D, after which the molecules were obtained in the .sdf format, which was later formatted as the ligand of the study and converted into the pdbqt format employing the Open Babel tool (Alzarea et al., 2025). On the other hand, a standard molecule such as diclofenac was derived from the online database of NCBI, “Pubchem” (https://pubchem.ncbi.nlm.nih.gov/). The molecule was further processed through Open Babel via PyRx for molecular docking and simulation.

#### 2.3.3 Performing the proteins–ligand molecular docking

The processed proteins’ crystal structures were uploaded into the *PyRx Python Prescription 0.8* (https://pyrx.sourceforge.io/) and converted into the pdbqt format as a macromolecule of the study (Dallakyan and Olson, 2014). The ligands and diclofenac .sdf file were uploaded through the “Open Babel” and were processed into the pdbqt format for producing a ligand molecule for molecular docking after energy minimization. Molecular docking was performed by creating a grid dimension selecting the complete protein and selecting 20 core CPUs with effectiveness, and the final docked molecules were saved for further processing. BIOVIA Discovery Studio was preferred to analyze the results obtained after molecular docking, including the 2D structures of proteins’ and ligands’ interactions, protein–ligand interactions, and pocket identification.

PyRx, which combines AutoDock Vina, AutoDockTools for Windows, and Open Babel, was used to perform the docking. The ligands for AB 12 were prepared using ChemDraw, and the structure of diclofenac was obtained as an SDF file from PubChem and changed into PDBQT with Gasteiger charges and AutoDock atom types. Using BIOVIA Discovery Studio, the protein (COX-2) was cleaned and protonated. Then, using PyRx’s macromolecule workflow, which also uses Gasteiger charges and AutoDock atom types, they were changed to the PDBQT format. A grid box with dimensions in Å was prepared for molecular docking against COX-2. The box was centered on the cyclooxygenase-2 active site and included nearby residues to allow the ligand to move freely. Docking developed each ligand in 10 poses, and the best one based on the affinity score was selected for further MD simulation. Docking used AutoDock Vina’s empirical scoring function, which includes steric, hydrogen bonding, and hydrophobic interactions to develop the binding free energy (kcal/mol). The best pose with the least amount of energy was chosen. Vina uses a simplified empirical model particularly defined as a semi-empirical force field. The docking was carried out in AutoDock Vina (PyRx) with an exhaustiveness score of 20, which is higher than the default score of 8. This made sure that the COX-2 binding site was sampled more deeply in terms of its shape, which made the predicted binding modes more reliable, utilizing a stochastic global search algorithm integrated with local optimization. So, even though the genetic algorithm parameters are not applicable in Vina, the higher exhaustiveness score helped perform a thorough search.

#### 2.3.4 Preparing the enzyme—COX-2, diclofenac, and ligand AB 12—for molecular dynamic simulation

Pymol application was preferred for converting the .dsv files into .pdb files and named as complex post molecular docking, where the separately imported protein and ligand files after molecular docking were joined through Pymol and were saved in the form of a .pdb file for performing the MD simulation by means of GROMACS (https://www.gromacs.org/) (Abraham et al., 2015), and the results were visualized through visual molecular dynamics (VMD) (Humphrey et al., 1996). After MD simulation, various aspects of simulations like development of root-mean-square deviation (RMSD), radius of gyration, and root-mean-square fluctuations (RMSF) were developed to analyze the outcomes of MD simulation of the protein and novel ligand complex in order to describe the interactions, which would help in determining their stability during stimulatory interactions.

MD simulations were performed using the CHARMM27 force field and the TIP3P water model, and simulations were run with the protein and ligand prepared in chimera with Gasteiger charges. The system was subjected to steepest-descent energy minimization (50,000 steps, convergence at maximum force <1,000 kJ/mol) followed by NVT equilibration to stabilize the temperature and NPT equilibration to stabilize the pressure and density. After which, a 50-ns production MD run was performed using the leap-frog integrator with a 2-fs timestep. Throughout the run, three-dimensional periodic boundary conditions (pbc = xyz) with isotropic pressure coupling were applied. The periodic box was rectangular/orthorhombic with dimensions of 9.794 nm × 8.294 nm × 8.421 nm after NVT, 9.756 nm × 8.262 nm × 8.388 nm after NPT, and 9.781 nm × 8.283 nm × 8.410 nm at the start of production MD, as recorded in the respective simulation files.

#### 2.3.5 Studying the binding free energies through MM–PBSA and MM–GBSA analyses

The current study utilized the molecular mechanics generalized born surface area (MM–GBSA) and molecular mechanics Poisson–Boltzmann surface area (MM–PBSA) methods to identify the binding free energies and per-residue contributions in the molecular systems for both simulations, i.e., COX-2 with ligand AB-12 and COX-2 with ligand diclofenac. The generalized born solvent model was preferred for the MM–GBSA calculations, and GBSA = 2 was the parameter used to detect the non-polar solvation energies. Using these settings including the interactions in the internal energy calculations, we were able to obtain internal, van der Waals, electrostatic, polar solvation, and non-polar solvation energy components. MM–PBSA analysis was performed by using the Poisson–Boltzmann model to determine solvation effects in a more accurate manner. The output was drawn into an Excel spreadsheet, with each row placed with a different residue and the columns dedicated for the values of average energy, standard deviations, and standard error of mean. This structured format made it easy to observe and compare the energy component contributions at the residue level, which helped us better understand molecular interactions and stability. The study provides a better outlook into the key residues involved in the interactions and further supports the drug designs and statistical compilation of the data for a better understanding. A higher negative value expresses favorable protein and ligand binding (Alzarea et al., 2025).

### 2.4 *In vivo* experimentation

#### 2.4.1 Animal experimental ethics and the determination of acute oral toxicity and test dose

Albino mice of either sex were selected to perform the acute oral toxicity analysis of the five synthetic compounds. The mice were kept under standard conditions and were purchased a week prior to the beginning of experiment for laboratory acclimatization, during which period the 12-h dark and light cycle was maintained with a constant temperature of 25 °C ± 2 °C with sufficient supply of water. Different but calculated doses of the chemicals were administered to the group of six mice, for each molecule, to determine the lethal dose (LD50). The oral LD50 values were converted into parenteral LD50 (Oecd, 2009; Jacob et al., 2022), and one-fifth of the determined parenteral LD50 values were selected as the test dose of the drug against Eddy’s hot plate and tail immersion methods of algesia induction (Wang et al., 2015; Rispin et al., 2002) (Table 1).

**TABLE 1:** Protein–ligand docking results for COX-1 and COX-2 with synthetic compounds and diclofenac.

Name of protein	Name of the ligand	Highest affinity score kcal/Mol	Ionic or van der Waals’s interactions (Å units)	Distance, Å; amino acids involved in hydrogen bonds
COX-1	AB 11	−9.2	ASN A: 382, PHE A: 210, HIS A: 207, TYR A: 285, TRP A: 387, LEU A: 390, ALA A: 199, MET A: 391, GLN A: 203, VAL A: 447	2.75; THR A: 206
	AB 12	−9.3	ASN A: 382, HIS A: 207, PHE A: 210, THR A: 206, TYR A: 285, TRP A: 387, ALA A: 199, LEU A: 390, GLN A: 203, VAL A: 447	-
	AB 13	−8.8	TRP A: 387, THR A: 206, HIS A: 207, VAL A: 447, GLN A: 203, HIS A: 388, PHE A: 210, MET A: 391, ASN A: 382	3.07; TYR A: 385
	AB 14	−9.1	VAL A: 447, ASN A: 382, HIS A: 207, PHE A: 210, THR A: 206, TYR A: 385, TRP A: 387, ALA A: 199, LEU A: 390, GLN A: 203	-
	AB 15	−9.0	GLY A: 45, GLN A: 44, GLN A: 461, TYR A: 39, PRO A: 40, VAL A: 155, ASP A: 135, TYR A: 136, ILE A: 46	2.15; SER A: 154
	Diclofenac	−7.5	ASP A: 135, PRO A: 156, TYR A: 130, SER A: 154, GLY A: 45, LEU A: 152, PRO A: 40, TYR A: 39, GLN A: 461	2.41; CYS A: 41
COX-2	AB 11	−9.5	ALA A: 199, LEU A: 390, TRP A: 387, THR A: 206, PHE A: 210, ASN A: 382, GLN A: 203	1.99; TYR A: 385 2.40; HIS A: 388
	AB 12	−9.6	ARG A: 44, LEU A: 152, VAL A: 155, GLY A: 135, GLY A: 45, CYS A: 36, CYS A: 47, VAL A: 46, TYR A: 130, TYR A: 136, MET A: 48	2.88; HIS A: 39 3.03; GLN A: 461 1.90; PRO A: 154
	AB 13	−9.5	ASN A: 382, PHE A: 210, TYR A: 285, TRP A: 387, LEU A: 390, PHE A: 200, LEU A: 391, GLN A: 203, ALA A: 199, VAL A: 447	2.61; HIS A: 207 2.87; THR A: 206 2.84; HIS A: 388
	AB 14	−10	GLN A: 203, ALA A: 199, LEU A: 390, ALA A: 202, TRP A: 387, THR A: 206, PHE A: 210, ASN A: 382	2.38; HIS A: 388 1.90; TYR A: 385
	AB 15	−9.4	PRO A: 156, GLY A: 135, VAL A: 46, TYR A: 130, GLY A: 45, CYS A: 41, HIS A: 39, GLN A: 42, LYS A: 468, GLU A: 465	-
	Diclofenac	−7.1	TYR A: 130, VLA A: 46, CYS A: 47, LYS A: 468, GLU A: 465, PRO A: 153, ARG A: 44	1.98; HIS A 39 2.11; GLN A 461 2.52; CYS A 41 2.51; GLY A 45

The following section describes the approach used to assess the analgesic activity of the five molecules against Eddy’s hot plate and tail immersion method of algesia induction in rats; furthermore, the study aimed to evaluate the efficacy of the five molecules in preventing Eddy’s hot plate and tail immersion methods of algesia. The experimental design involved the administration of different doses of the test substances to groups of rats prior to induce algesia, as mentioned above. The animals were observed for pain severity, which was scored using a standardized method. The results showed that some of the substances tested had significant analgesic activity against both the models, while others were effective only against one type. Overall, this approach helps in the development of potential analgesic agents and could contribute to the development of novel therapeutics for the management of pain (Walum, 1998; Kaplancikli et al., 2011; Fan et al., 2014).

#### 2.4.2 Analgesic activity assessment in rats

##### 2.4.2.1 Animal groups

Seven groups of six mice were chosen. A standard medication called diclofenac was administered to one group of mice (standard). Another group was treated only with distilled water, the individuals of which were previously treated with carrageenan because they were considered the diseased models for reference. Synthetic substances were used to treat the remaining five groups.

##### 2.4.2.2 Groups of animals for determining the analgesic activity

Group I: Normal animals + induced algesia (diseased control group).
Group II: Diclofenac + induced algesia (standard group).
Group III: AB11 (Molecule 1) + induced algesia.
Group IV: AB13 (Molecule 2) + induced algesia.
Group V: AB12 (Molecule 3) + induced algesia.
Group VI: AB14 (Molecule 4) + induced algesia.
Group VII: AB15 (Molecule 5) + induced algesia.

#### 2.4.3 Anti-inflammatory activity in rats

##### 2.4.3.1 Animal groups

Seven groups of six rats were chosen. A standard medication named diclofenac was administered to one group of rats (standard). Another group was treated only with distilled water because they were considered the diseased models for reference. Synthetic substances were used to treat the remaining five groups (Alqarni et al., 2024).

##### 2.4.3.2 Groups of animals for determining anti-inflammatory activity

Group I: Normal animals + carrageenan induced paw edema (diseased control group).
Group II: Diclofenac + carrageenan induced paw edema (standard group).
Group III: AB11 (Molecule 1) + carrageenan induced paw edema.
Group IV: AB13 (Molecule 2) + carrageenan induced paw edema.
Group V: AB12 (Molecule 3) + carrageenan induced paw edema.
Group VI: AB14 (Molecule 4) + carrageenan induced paw edema.
Group VII: AB15 (Molecule 5) + carrageenan induced paw edema.

#### 2.4.4 Compound administration

The rats received oral doses of the synthetic compounds and diclofenac to counter the algesia induced with the above-described models. Based on the lethal dose (LD50) values, the dosage of each component was established and modified as necessary.

#### 2.4.5 Statistical analysis

To ascertain the effects of each molecule against Eddy’s hot plate and tail immersion methods of induced algesia, the data obtained from the behavioral recordings were statistically analyzed. Statistical comparisons were made between the synthetic compound group (test group), the standard diclofenac group, and the control group by using GraphPad Prism statistical software (version v.9.5.1). Data were processed through a two-way ANOVA and one-way ANOVA (anti-inflammatory) using Dunnett’s test as a multiple comparison test for attaining significance. The values of the groups are represented as mean ± SEM; with values less than or equal to 0.05 considered statistically significant and those less than or equal to 0.01 considered highly significant (Festing, 2002; Gosselin, 2018).

### 2.5 Ethical approval

Prior to the *in vivo* experimentation, the proposal was submitted to the institutional ethical committee describing in detail the purpose and rationale of performing the experiments on the animal models, and the animal experiments were started only after obtaining the approval bearing number RES-2025–077.

## 3 Results

### 3.1 Synthesis of the five novel isatin hydrazone derivatives

**FIGURE 3:**
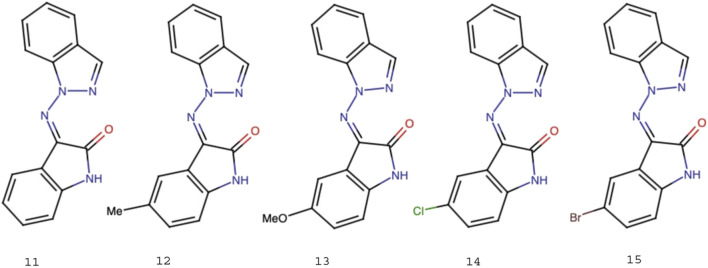
Five Schiff bases obtained following the synthetic procedure.

Five isatin derivatives (8), namely, isatin, methylisatin, methoxyisatin, chloroisatin, and bromoisatin, were allowed to react with 1-aminoindazole (9) by refluxing ethanol to afford the corresponding Schiff bases 11, 12, 13, 14, and 15, respectively, in very good yields (Figure 3). All the products were characterized by IR, NMR, mass spectrometry, elemental analysis, and melting point analysis.

#### 3.1.1 3-(Indazole-1-ylimino)-1H-indol-2-one (R = H) (11)

(Yield 82%); m.p. 325–327 C dec.; IR: ν_max_/cm^-1^ 3,134, 1712, 1,680, 1,579, 1,458, 1,328, 1,220, 1,188, 1,041, 750; ^1^H NMR (DMSO, 500 MHz): δ 10.94 (s, 1H), 9.02 (d, 1H), 8.67 (s, 1H), 7.94 (dd, 2H), 7.65 (t, 1H), 7.44 (m, 2H), 7.08 (t, 1H), 6.93 (d, 1H); ^13^C NMR (DMSO, 500 MHz): δ 165.2, 145,7, 139.9, 137.5, 136.9, 133.6, 129.8, 129.1, 124.2, 123.7, 121.9, 121.7, 115.7, 111.5, 110.7; MS: *m/z* 263 (MI^+^), 203, 163, 153. Anal. Calcd. for C_15_H_10_N_4_O: C, 68.70; H, 3.82; N, 21.37. Found: C, 68.57; H, 3.90; N, 21.48.

#### 3.1.2 3-(Indazole-1-ylimino)-1H-indol-2-one (R = Me) (12)

(Yield 84%); m.p. 292–294 C dec.; IR: ν_max_/cm^-1^ 3,170, 3,095, 2,916, 1712, 1,676, 1,577, 1,477, 1,421, 1,301, 1,172, 1,041, 767; ^1^H NMR (DMSO, 500 MHz): δ 11.07 (s, 1H), 9.07 (s, 1H), 8.76 (s, 1H), 7.94 (dd, 2H), 7.67 (t, 1H), 7.46 (m, 2H), 6.96 (d, 1H), 2.06 (s, 3H); ^13^C NMR (DMSO, 500 MHz): δ 164.9, 144.4, 140.1, 138.5, 137.9, 135.8, 132.5, 129.4, 129.1, 125.6, 124.5, 123.8, 122.1, 117.1, 112.1, 111.6; MS: *m/z* 277 (MI^+^), 249, 221, 159, 118. Anal. Calcd. for C_16_H_12_N_4_O: C, 69.57; H, 4.35; N, 20.29. Found: C, 69.72; H, 4.26; N, 19.95.

#### 3.1.3 3-(Indazole-1-ylimino)-1H-indol-2-one (R = OMe) (13)

(Yield 81%); m.p. 256–258 C dec.; IR: ν_max_/cm^-1^ 3,170–2,900, 1708, 1,674, 1,577, 1,477, 1,307, 1,213, 1,174, 1,045, 750; ^1^H NMR (DMSO, 500 MHz): δ 9.89 (s, 1H), 7.85 (d, 1H), 7.83 (s, 1H), 7.50 (dd, 2H), 6.80 (t, 1H), 6.60 (t, 1H), 6.20 (dd, 1H), 5.98 (d, 1H), 2.92 (s, 3H); ^13^C NMR (DMSO, 500 MHz): δ 165.4, 154.4, 139.9, 139.3, 137.8, 136.9, 129.1, 124.2, 123.7, 121.9, 118.6, 116.3, 116.2, 111.5, 111.0, 55.6; MS: *m/z* 293 (MI^+^), 261, 247, 149. Anal. Calcd. for C_16_H_12_N_4_O_2_: C, 65.75; H, 4.12; N, 19.17. Found: C, 65.51; H, 4.21; N, 19.31.

#### 3.1.4 3-(Indazole-1-ylimino)-1H-indol-2-one (R = Cl) (14)

(Yield 79%); m.p. 330–332 C dec.; IR: ν_max_/cm^-1^ 3,230, 3,100, 1726, 1,695, 1,614, 1,577, 1,458, 1,421, 1,301, 1,184, 1,050, 748; ^1^H NMR (DMSO, 500 MHz): δ 11.10 (br.s, 1H), 9.06 (s, 1H), 8.75 (s, 1H), 7.94 (dd, 2H), 7.58 (dd, 2H), 6.93 (m, 2H); ^13^C NMR (DMSO, 500 MHz): δ 159.2, 149.3, 137.9, 137.3, 129.4, 124.5, 122.1, 113.9. MS: *m/z* 297 (MI^+^), 269, 234, 118. Anal. Calcd. for C_15_H_9_ClN_4_O: C, 60.71; H, 3.03; N, 18.89. Found: C, 60.48; H, 3.17; N, 18.77.

#### 3.1.5 3-(Indazole-1-ylimino)-1H-indol-2-one (R = Br) (15)

(Yield 83%); m.p. 337–339 C; IR: ν_max_/cm^-1^ 3,223, 3,100, 1726, 1,695, 1,612, 1,577, 1,458, 1,420, 1,300, 1,267, 1,184, 1,045, 740; ^1^H NMR (DMSO, 500 MHz): δ 11.10 (br.s, 1H), 9.20 (s, 1H), 8.70 (s, 1H), 7.92 (dd, 2H), 7.65 (m, 2H), 7.43 (t, 1H), 6.9 (d, 1H); ^13^C NMR (DMSO, 500 MHz): δ 165.8, 144.7, 140.1, 137.9, 135.7, 135.5, 131.8, 129.4, 124.5, 123.8, 122.1, 117.6, 113.3, 112.7, 111.6; MS: *m/z* 342 (MI^+^), 313, 285, 235, 119. Anal. Calcd. for C_15_H_9_BrN_4_O: C,52.78; H, 2.64; N, 16.42. Found: C, 52.63; H, 2.78; N, 16.33.

### 3.2 Molecular docking and molecular dynamic simulations

**FIGURE 4:**
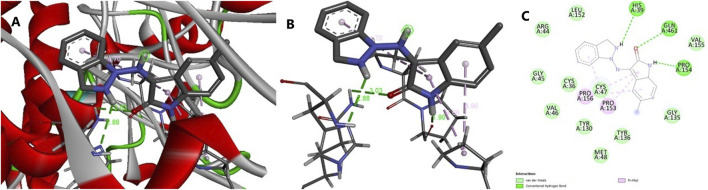
Molecular docking results of protein COX-2 and ligand AB 12. Figure 4. Interaction between the ligand (synthetic molecule AB 12) and the enzyme protein cyclooxygenase-2 (COX-2). **(A)** Receptor interaction with the ligand. **(B)** Protein interactive pocket atoms with the ligand. **(C)** 2D illustration of amino acids interacting with the ligand through hydrogen bonds, van der Waals forces, and other interactions.

**FIGURE 5:**
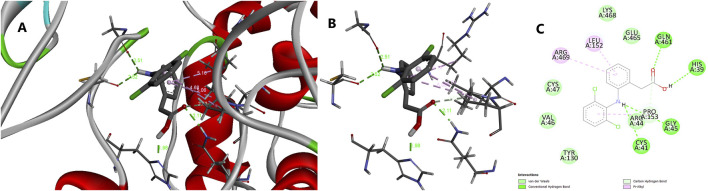
Molecular docking results of protein COX-2 and ligand diclofenac (Figure 6). Interaction between the ligand (diclofenac) and the enzyme protein COX-2. **(A)** Receptor interaction with the ligand. **(B)** Protein interactive pocket atoms with the ligand. **(C)** 2D illustration of amino acids interacting with the ligand through hydrogen bonds, van der Waals forces, and other interactions.

**FIGURE 6:**
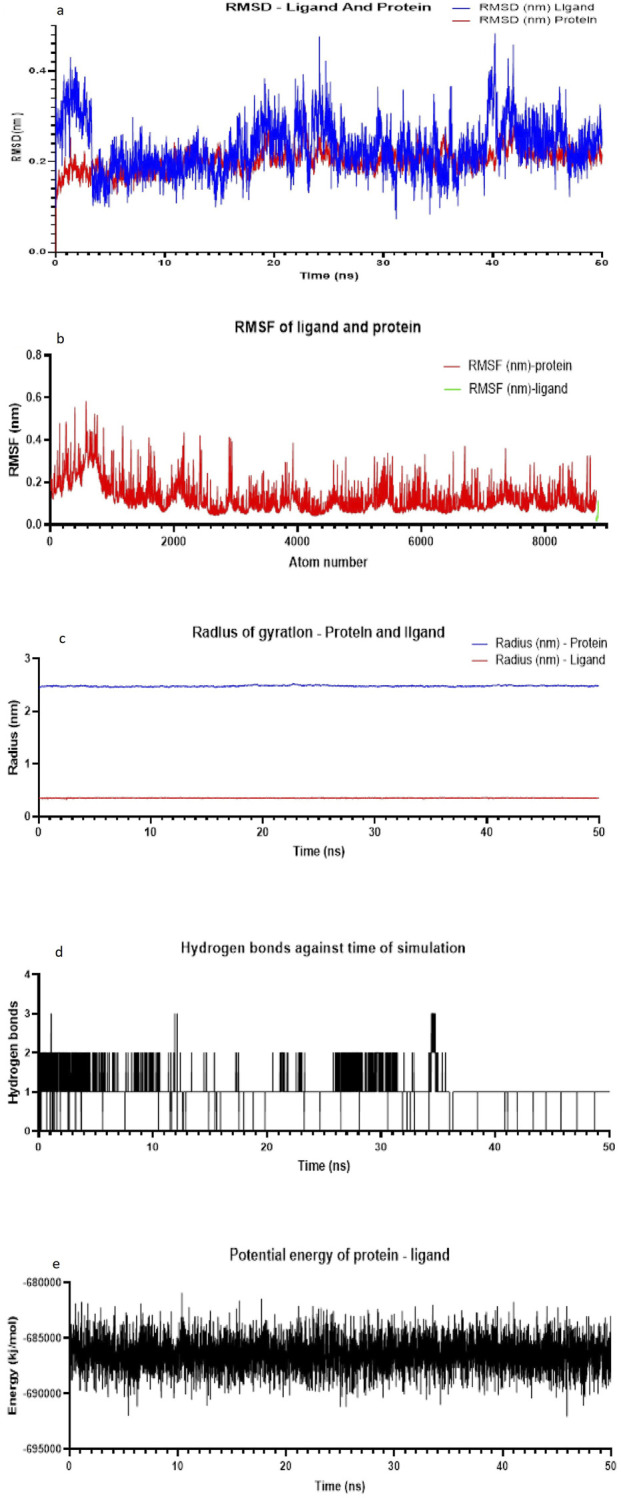
Structural and energetic stability of (COX-2/AB 12) over the course of the (50-ns) MD simulation. **(a)** Root-mean-square deviation (RMSD) of the protein atoms and ligand atom relative to the time (ns). A stable trajectory is indicated by the convergence to a plateau approximately 0.1 nm–0.4 nm after 50 ns. **(b)** Root-mean-square fluctuation (RMSF) per residue for the protein atoms. Peaks indicate regions of high flexibility, such as loops and terminals. Notably, the binding site shows low flexibility. **(c)** Radius of gyration (Rg) of the protein, measuring its overall firmness. A stable Rg value of approximately 2.5 nm for the protein and 0.4 nm for the ligand suggests the maintenance of a globular, folded structure. **(d)** Number of intramolecular hydrogen bonds within the protein over time. The system maintains an average of two hydrogen bonds, consistent with a stable folded state. **(e)** Potential energy of the simulated system. The stability and low fluctuation of the energy confirm that the simulation reached equilibrium and was thermodynamically stable. **(a)** RMSD plot of the protein (COX-2) and ligand (AB 12). **(b)** RMSF plot of the protein (COX-2) and ligand (AB 12). **(c)** Radius of gyration of the protein (COX-2) and ligand (AB 12). **(d)** Number of hydrogen bonds against the time of simulation (COX-2) and ligand (AB 12). **(e)** Potential energy of the protein and ligand in a simulation system (COX-2) and ligand (AB 12).

**FIGURE 7:**
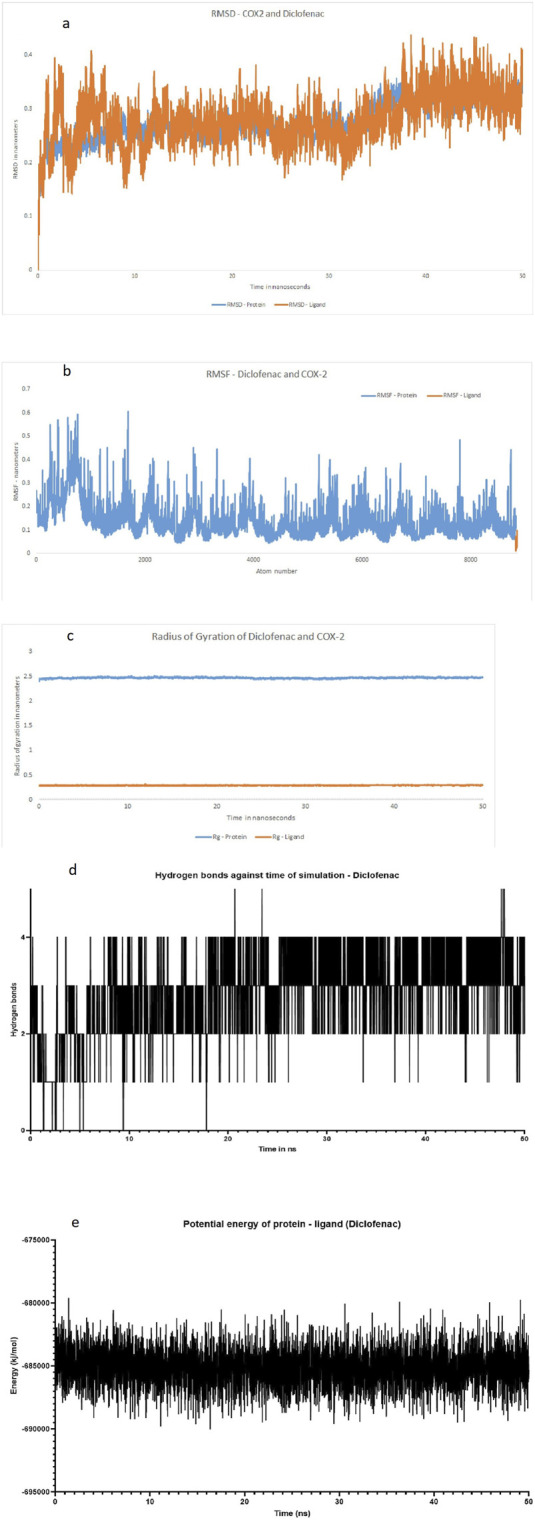
Structural and energetic stability of (COX-2/diclofenac) over the course of the (50-ns) MD simulation. **(a)** Root-mean-square deviation of the protein atoms and diclofenac atom relative to the time (ns). **(b)** Root-mean-square fluctuation per residue for the protein atoms. Peaks indicate regions of high flexibility, such as loops and terminals. Notably, the binding site shows low flexibility. **(c)** Radius of gyration (Rg) of the protein, measuring its overall firmness. A stable Rg value of approximately 2.5 nm for the protein and 0.3 nm–0.4 nm for the ligand suggests the maintenance of a globular, folded structure. **(d)** Number of intramolecular hydrogen bonds within the protein over time. The system maintains a range of 1–5 hydrogen bonds, consistent with a stable folded state. **(e)** Potential energy of the simulated system. The stability and low fluctuation of the energy confirm that the simulation reached equilibrium and was thermodynamically stable. **(a)** RMSD plot of the protein (COX-2) and ligand (diclofenac). **(b)** RMSF plot of the protein (COX-2) and ligand (diclofenac). **(c)** Radius of gyration of the protein (COX-2) and ligand (diclofenac). **(d)** Number of hydrogen bonds against the time of simulation, protein (COX-2), and ligand (diclofenac). **(e)** Potential energy of the protein and ligand in a simulation system, protein (COX-2), and ligand (diclofenac).

The molecular docking results for compounds AB11–AB15 against the COX enzymes are summarized in Table 1 and illustrated in Figure 4. The ligand AB 12 interacted with cyclo-oxygenase-2 at a hydrogen bond length of 2.88., 3.03, and 1.90 Å with HIS A: 39, GLN A: 461, and PRO A: 154 amino acids, respectively, expressing one of the best binding scores of −9.6 kcal/mol. Furthermore, the interaction between COX-2 and the ligand was formed even through van der Waals interaction with ARG A: 44, LEU A: 152, VAL A: 155, GLY A: 135, GLY A: 45, CYS A: 36, CYS A: 47, VAL A: 46, TYR A: 130, TYR A: 136, and MET A: 48 amino acids. Another ligand molecule, AB 15, also expressed similar results with the best affinity score of – 10 kcal/mol with the COX-2 protein; however, fewer hydrogen bonds were observed with van der Waals interactions with only seven amino acids. Ligand AB 12 was chosen as the best ligand because of two reasons: the best affinity score and more van der Waals interactions. AB 14 was also identified with better interactions but with a lower affinity score of −9.4 kcal/mol and various amino acid interactions, which can be considered next only to AB 12, but with lower values (Table 1) (Figure 4). The diclofenac interacted with cyclo-oxygenase-2 at a hydrogen bond length of 1.98, 2.11, 2.52, and 2.51 Å with HIS A: 39, GLN A: 461, CYS A 41, and Gly A 45 amino acids, respectively, expressing the best binding scores of −7.1 kcal/mol. Furthermore, the interaction between COX-2 and the ligand was formed even through van der Waals interaction with TYR A: 130, VLA A: 46, CYS A: 47, LYS A: 468, GLU A: 465, PRO A: 153, ARG A: 44, and other amino acids. With COX-1, the hydrogen bond was established between the CYS A: 41 of COX-1 at 2.41 Å and diclofenac (Table 1) (Figure 5).The MD simulation of the best pose obtained through molecular docking of COX-2 and ligand AB 12 was simulated in virtual cellular conditions using GROMACS. The structural and energetic stability of the COX-2/AB 12 was well-established through the results of simulations over the course of 50 ns. The RMSD results obtained for protein and ligand atoms relative to time showed data well within the range, i.e., ligand: 0.073864–0.4818 and protein: 0.000502–0.323969. A stable trajectory was evident ranging between 0.1 nm and 0.4 nm for 50 ns. RMSF for the complete atoms of the protein and ligand expressed a stable binding site and showed low fluctuations, indicating stability throughout the 50-ns simulation. The radius of gyration of the protein and ligand, i.e., 2.5 nm for the protein and 0.4 nm for the ligand, suggests the maintenance of a globular, folded, stable structure. The hydrogen bonds ranged between 0 and 3 for the system throughout the simulation, expressing a stable folded structure and compact binding site with a low consistent potential energy. The results indicate that the protein–ligand complex was stable and folded with the lowest possible energy in a system, highlighting that the simulation reached equilibrium and was thermodynamically stable (Table 2; Figure 6). The MD simulation of the best pose obtained through molecular docking of COX-2 and diclofenac was performed. The structural and energetic stability of the COX-2/diclofenac was well-established through the results of simulations over the course of 50 ns. The RMSD results obtained for the protein and ligand atom relative to time showed data well within the range, i.e., ligand: 0.0004973–0.4365657 and protein: 0.0005027–0.3684722. RMSF for the complete atoms of protein and ligand expressed a stable binding site and showed low fluctuations, indicating stability throughout simulation of 50 ns. The radius of gyration of the protein and ligand, i.e., 2.5 nm for the protein and 0.3 nm for the ligand, suggests the maintenance of a globular, folded, stable structure. The hydrogen bonds ranged between 0 and 5 for the system throughout the simulation, expressing a stable folded structure and compact binding site with low consistent potential energy. The results indicate that the protein–ligand complex was stable and folded with lowest possible energy in a system, highlighting that the simulation reached equilibrium and was thermodynamically stable (Table 2; Figure 7).

**TABLE 2:** Molecular dynamic (MD) simulation of the ligands (AB 12 and diclofenac) and COX-2.

Protein	Ligand	Root-mean-square-deviation (RMSD) nm	Radius of gyration (Rg) nm	Root-mean-square-fluctuations (RMSF) nm	Potential energy of the system kj/Mol	Hydrogen bonds
COX-2	AB 12	Ligand: 0.073864–0.4818 Protein: 0.000502–0.323969	Protein: 2.402837–2.53762 Ligand: 0.319048–0.371637	Protein: 0.0417–0.5821 Ligand: 0.0153–0.1124	−692039 to −680924	0–3
COX-2	Diclofenac	Ligand: 0.0004973 −0.4365657 Protein: 0.0005027–0.3684722	Protein: 2.403864 −2.514971 Ligand: 0.284675–0.316297	Protein: 0.046–0.6045 Ligand: 0.0124–0.0989	−689986 to −679629.75	0–5

The total energy decomposition data of the complex of COX-2 with both the ligands (AB 12 and diclofenac) were studied using MM–PBSA and MM–GBSA. Regarding the AB-12 ligand, the residue energy decomposition analysis shows that the ligand (L:B:LIG:584) is a major contributor to binding because of its very low total energy (16.73 kcal/mol). Amino acid residues of proteins that are involved in the interaction, such as HIS39, PRO153, PRO154, and LEU152, contribute to protein stability with their very low total energies. Charged residues of proteins such as ARG44, LYS137, and ARG469 display a characteristic salt bridge formation, where substantially favored electrostatic interactions are nearly completely counterbalanced by equally significant unfavorable polar desolvation penalties (Table 3). The obtained binding free energy (ΔG_bind) for the protein–ligand complex is +2.54 kcal/mol, which suggests unfavorable binding interaction. This results due to two opposing energies, which almost completely cancel each other out. However, it also involves a very favorable gas-phase interaction energy (ΔGGAS = −82.35 kcal/mol) and a very high solvation penalty (ΔGSOLV = +84.89 kcal/mol). Breaking down the energy in the gas phase shows that both van der Waals (ΔVDWAALS = −32.19 kcal/mol) and electrostatic (ΔEEL = −50.16 kcal/mol) forces play a substantial role in the fundamental affinity. The polar solvation penalty (ΔEGB = +89.09 kcal/mol) is by far the most important as it works against the binding of the protein and ligand. The nonpolar solvation (ΔESURF = −4.20 kcal/mol) adds less favorability (Table 4).

**TABLE 3:** AB 12 and COX-2 MM–PBSA results: total energy decomposition data of the complex (AB 12–COX-2).

Residue	Internal (avg)	Internal (std. dev.)	van der Waals (avg)	van der Waals (std. dev.)	Electrostatic (avg)	Electrostatic (std. dev.)	Polar solvation (avg)	Polar solvation (std. dev.)	Nonpolar solv. (avg)	Nonpolar solv. (std. dev.)	Total (avg)	Total (std. dev.)
R:A:ASN:34	0	0.811051	−0.294	1.467849	23.3705	5.044692	−23.004	4.506769	−0.0494	0.034375	0.023097	6.969465
R:A:PRO:35	0	0.595384	−0.071	1.031294	−0.563	0.937662	0.5885	0.792315	0	0.041564	−0.0455	1.710774
R:A:CYS:36	0	2.186374	−0.9665	0.982658	−1.8985	2.048687	2.1965	0.324267	−0.13184	0.043289	−0.80034	3.170172
R:A:CYS:37	0	2.895602	−0.1455	0.48533	−1.75	3.035248	1.911	1.248042	0	0.02151	0.0155	4.403505
R:A:SER:38	0	0.526087	−0.0595	0.516	−0.7415	4.684022	0.8505	2.417179	0	0.052994	0.0495	5.322467
R:A:HIS:39	0	2.857419	−0.266	0.263463	−8.231	5.778071	4.499	2.543804	−0.1324	0.083216	−4.1304	6.935288
R:A:PRO:40	0	0.690136	−0.1525	0.077403	1.054	2.340472	−1.5365	1.657267	0	0.029284	−0.635	2.950844
R:A:CYS:41	0	0.526795	−0.2385	0.088956	−2.385	0.628667	2.527	1.184578	−0.06085	0.006596	−0.15735	1.443578
R:A:GLN:42	0	2.281126	−0.092	0.220074	−2.228	3.892196	2.2435	1.4915	0	0.099497	−0.0765	4.757692
R:A:ARG:44	0	2.172939	−1.063	0.626313	38.5435	22.64345	−37.181	19.06552	−0.22647	0.383323	0.073028	29.68974
R:A:GLY:45	0	1.019648	−0.42	0.251645	−6.8005	3.523455	6.719	2.176266	−0.0966	0.014208	−0.5981	4.272479
R:A:VAL:46	0	1.001263	−0.486	0.884933	2.1995	1.504181	−1.5835	0.518938	−0.0393	0.125747	0.090695	2.081659
R:A:CYS:47	0	0.084146	−0.954	0.579245	1.93	2.440378	−0.456	1.260178	−0.18348	0.022554	0.337015	2.80831
R:A:MET:48	0	1.197132	−0.2975	0.213984	−0.3465	3.698092	0.297	0.884485	−0.0215	0.128606	−0.3685	3.994203
R:A:SER:49	0	0.774989	−0.226	0.159049	−1.216	1.404977	1.3535	0.472553	−0.03151	0.028348	−0.12001	1.680469
R:A:TYR:55	0	6.144051	−0.045	0.662776	1.63	0.362355	−1.6005	1.56578	0	0.131164	−0.0155	6.386611
R:A:TYR:130	0	3.270369	−0.5545	2.063691	−0.1035	1.109818	0.298	0.657615	−0.0714	0.054547	−0.4314	4.076918
R:A:TYR:134	0	0.888126	−0.0715	2.056026	−0.1375	3.133564	0.2615	1.153029	0	0.179891	0.0525	4.024559
R:A:GLY:135	0	2.027982	−0.749	0.793544	−2.431	2.002926	2.9385	1.485976	−0.16083	0.101527	−0.40233	3.312487
R:A:TYR:136	0	8.417399	−0.453	0.509771	0.027	1.745802	0.0455	0.157915	−0.08483	0.065928	−0.46533	8.613337
R:A:LYS:137	0	3.017932	−0.076	0.562873	29.873	16.61462	−29.611	16.76301	0	0.166929	0.186	23.80119
R:A:ALA:151	0	0.101823	−0.0805	0.23575	−1.1185	3.969898	1.211	2.238676	−0.00119	0.009076	0.010812	4.564842
R:A:LEU:152	0	0.363453	−1.076	0.58834	2.6375	1.624106	−2.4275	1.016315	−0.16569	0.004158	−1.03169	2.036879
R:A:PRO:153	0	3.587153	−2.196	1.154707	−1.142	1.846812	0.9255	1.29936	−0.20113	0.016341	−2.61363	4.393214
R:A:PRO:154	0	2.269106	−0.5225	0.358966	−5.449	3.259529	4.5725	2.28219	−0.02544	0.035521	−1.42444	4.594767
R:A:VAL:155	0	3.024296	−0.4885	2.23773	1.15	5.755299	−0.9825	0.911003	−0.01187	0.021288	−0.33287	6.935967
R:A:PRO:156	0	2.739332	−0.815	1.526662	1.391	1.758257	−1.5755	1.400449	−0.14296	0.09775	−1.14246	3.859652
R:A:GLN:461	0	0.20294	−0.981	1.385483	−2.876	0.944241	3.109	0.616601	−0.07475	0.032656	−0.82275	1.798221
R:A:GLU:465	0	3.797871	−0.4805	0.262943	−54.6915	10.06351	54.17	11.31062	−0.10649	0.065566	−1.10849	15.61094
R:A:LYS:468	0	5.567052	−0.113	1.411139	39.238	9.896265	−38.73	10.9822	−0.02286	0.10639	0.372144	15.86
R:A:ARG:469	0	0.761554	−0.553	0.965263	32.802	3.07136	−32.4275	1.271623	−0.05304	0.037008	−0.23154	3.54448
L:B:LIG:584	0	2.419719	−16.095	0.918088	−25.0815	22.58692	61.145	27.30644	−3.24238	0.125246	16.72612	35.53202

**TABLE 4:** Energy component breakdown as binding energy analysis based on the MM–GBSA of protein (COX-2)–ligand (AB 12) complex.

Energy component	Average	SD (prop.)	SD	SEM (prop.)	SEM
ΔVDWAALS, van der Waals interaction energy	−32.19	1.67	2.44	1.18	1.72
ΔEEL (electrostatic interaction energy)	−50.16	31.43	31.43	22.22	22.22
ΔEGB (polar solvation energy (GB model))	89.09	33.64	33.64	23.79	23.79
ΔESURF (nonpolar solvation energy)	−4.2	0.35	0.43	0.25	0.31
ΔGGAS (gas phase interaction energy)	−82.35	31.47	33.86	22.25	23.95
ΔGSOLV (solvation free energy)	84.89	33.64	33.21	23.79	23.48
ΔTotal (total binding free energy)	2.54	46.07	0.66	32.57	0.47

As per the standard molecule diclofenac, the residue energy decomposition analysis identified various important residues for protein–ligand interaction. The ligand (LIG:584) diclofenac was the key component of the binding, with a total energy of −13.99 kcal/mol. This was due to very strong electrostatic (−18.96 kcal/mol) and van der Waals (−14.46 kcal/mol) interactions. Multiple amino acid residues such as HIS:39 (−4.65 kcal/mol) and LEU:152 (−1.85 kcal/mol) were also detected to be significant binding targets. Furthermore, HIS:39 was also a good binding point, which was mainly due to electrostatic forces (−7.13 kcal/mol), while LEU:152 was a good binding point due to both van der Waals (−1.32 kcal/mol) and electrostatic forces. GLN:461, with a strong electrostatic interaction (−3.87 kcal/mol), and VAL:46, with a strong van der Waals interaction (−1.24 kcal/mol), were other important contributors (Tables 5, 6).

**TABLE 5:** Diclofenac and COX-2 MM–PBSA results: total energy decomposition data of the complex (diclofenac–COX-2).

Residue	Internal (avg)	Internal (std. dev.)	van der Waals (avg)	van der Waals (std. dev.)	Electrostatic (avg)	Electrostatic (std. dev.)	Polar solvation (avg)	Polar solvation (std. dev.)	Nonpolar solv. (avg)	Nonpolar solv. (Std. dev.)	Total (avg)	Total (std. dev.)
R:A:CYS:36	1.842013165	1.3025	−0.4355	1.269381838	−0.218	1.85046292	0.405	0.743700545	−0.03325	0.016159285	−0.2817	2.996983045
R:A:CYS:37	3.591395342	2.5395	−0.0815	0.436662627	−0.167	1.62927775	0.284	0.787681725	0	0.009576489	0.0355	4.045228666
R:A:HIS:39	1.198545994	0.8475	−0.675	1.015025615	−7.125	2.11404789	3.359	0.389217163	−0.205675	0.037875625	−4.6467	2.662501186
R:A:PRO:40	0.96873629	0.685	−0.6475	0.452301061	−0.0945	1.03205438	0.2455	1.001115003	−0.000954	0.058733052	−0.4975	1.792719532
R:A:CYS:41	1.87595429	1.3265	−1.1035	1.245083632	−1.695	3.01385409	1.372	2.208695995	−0.13559	0.026316	−1.5621	4.362543385
R:A:GLN:42	3.02288149	2.1375	−0.761	0.456826006	−0.2565	4.03374829	0.385	2.123712904	−0.04802	0.048553888	−0.6805	5.489092979
R:A:ASN:43	0.127986327	0.0905	−0.2945	1.789472338	0.102	3.93589666	0.11	2.692673857	−0.0176	0.018296803	−0.1001	5.095164524
R:A:ARG:44	3.580081633	2.5315	−1.001	0.210870814	−1.4465	2.63853316	1.848	3.491211609	−0.126871	0.173688352	−0.7264	5.660568381
R:A:GLY:45	1.402899854	0.992	−0.5975	0.924025027	−1.3135	4.9858142	1.379	0.265748942	−0.125258	0.051490015	−0.6573	5.268165451
R:A:VAL:46	1.743018216	1.2325	−1.2395	1.396319179	0.049	2.06850248	0.2865	0.385349517	−0.07717	0.230444332	−0.9812	3.077031214
R:A:CYS:47	2.970555588	2.1005	−1.1135	0.111571726	−0.091	1.99715423	0.806	1.142968066	−0.161291	0.068654595	−0.5598	3.759835529
R:A:MET:48	0.317490945	0.2245	−0.071	0.061294372	−0.017	0.08340863	0.009	0.284850838	0	0.033545711	−0.079	0.440204288
R:A:THR:62	0.936209378	0.662	−0.025	0.29	−0.006	3.64160164	0.024	0.545179787	0	0.128226178	−0.007	3.812546846
R:A:TYR:130	5.84777308	4.135	−0.4385	0.415239991	−0.2745	4.6897368	0.5675	0.7694987	−0.056653	0.09864718	−0.2022	7.547474082
R:A:GLY:135	0.135764502	0.096	−0.108	0.213714763	−0.163	1.0697481	0.281	0.187619295	−0.023778	0.018512171	−0.0138	1.115352276
R:A:TYR:136	9.369871958	6.6255	−0.0525	0.858970459	0.0905	3.95026357	−0.0555	0.484728017	0	0.022569151	−0.0175	10.21628032
R:A:LYS:137	0.328804653	0.2325	−0.0485	1.239306762	−0.4395	7.8754042	0.531	4.314338999	0	0.082013639	0.043	9.071175902
R:A:LEU:152	1.518158259	1.0735	−1.319	0.417291864	−1.241	0.50908987	0.9635	0.842831092	−0.254628	0.023875185	−1.8511	1.857160137
R:A:PRO:153	1.433305445	1.0135	0.025	0.340023529	−0.1295	1.09031383	0.289	0.101872469	−0.180119	0.086818066	0.00438	1.837574523
R:A:PRO:154	0.567099639	0.401	−0.133	0.050089919	0.463	0.42812381	−0.2565	1.174929892	−0.002376	0.026362072	0.07112	1.374247506
R:A:VAL:155	0.943987553	0.6675	−0.1075	0.323524729	0.2255	1.6189451	−0.1885	1.971065511	0	0.016816131	−0.0705	2.73900457
R:A:PRO:156	0.335168614	0.237	−0.0945	0.914648156	0.016	0.82004939	0.004	0.164514437	−0.006689	0.08370807	−0.0812	1.286651581
R:A:GLN:461	2.935200249	2.0755	−0.083	0.867676495	−3.8735	3.32487507	2.457	1.571192222	−0.051091	0.010283507	−1.5506	4.784538431
R:A:GLU:465	0.013435029	0.0095	−0.9965	0.68458838	−1.8065	2.20033799	3.654	0.485962962	−0.063515	0.01877374	0.78749	2.355173338
R:A:TYR:466	5.468763846	3.867	−0.186	0.064548431	−0.159	2.84146133	0.2605	0.364185736	0	0.021275995	−0.0845	6.174020644
R:A:LYS:468	2.066166015	1.461	−0.6885	1.211707576	1.0145	0.25117375	−1.0515	0.557554706	−0.085075	0.011836642	−0.8106	2.472119102
R:A:ARG:469	2.453660531	1.735	−0.7815	1.518574414	−1.7965	4.90215833	1.516	2.3448114	−0.029542	0.012680094	−1.0915	6.152721007
L:B:LIG:584	1.356230806	0.959	−14.4645	2.05899059	−18.9585	1.18527138	22.5045	0.716436494	−3.076409	0.065666033	−13.995	2.828650876

**TABLE 6:** Energy component breakdown as binding energy analysis based on the MM–GBSA of protein (COX-2)–ligand (diclofenac) complex.

Energy component	Average	SD (prop.)	SD	SEM (prop.)	SEM
ΔVDWAALS, van der Waals interaction energy	−28.93	1.66	1.66	1.17	1.17
ΔEEL (electrostatic interaction energy)	−37.92	1.16	1.16	0.82	0.82
ΔEGB (polar solvation energy (GB model))	42.42	0.89	0.89	0.63	0.63
ΔESURF (nonpolar solvation energy)	−4.33	0.02	0.04	0.02	0.03
ΔGGAS (gas phase interaction energy)	−66.85	2.02	0.5	1.43	0.35
ΔGSOLV (solvation free energy)	38.1	0.89	0.93	0.63	0.66
ΔTotal (total binding free energy)	−28.75	2.21	1.43	1.56	1.01

### 3.3 Results of the preclinical study

The values of acute oral toxicity and calculated intraperitoneal test doses of the five coded molecules (AB11, AB13, AB12, AB14, and AB15) are given in Table 7. The oral LD_50_ value ranged between 2,100 mg/kg/mice– and 3,000 mg/kg/mice. For the safety of the test animals, the oral LD_50_ doses were multiplied with 0.27 (conversion factor), resulting in lower and safer LD_50_ doses. The test dose ED_50_ values actually used for efficacy studies are the one-fifth doses of the original LD_50_ values. Based on the body weight of the animals, the range of effective dose ED_50_ was calculated to be between 17 mg/kg– and 24 mg/kg for every individual mouse. The standard drug selected was diclofenac at a dose of 7.5 mg/kg.

**TABLE 7:** Acute oral toxicity and test dose.

Sr No	Name of the molecule	LD50 (oral)	Calculated LD50ip for IP route (LD50*0.27)	ED50 (1/5th of LD50 ip) – test dose	Dose per animal of 150 gm bodyweight (appx)
1	AB11	2,100 mg/kg	567 mg/kg	113.4 mg/kg	17 mgs
2	AB12	3,000 mg/kg	810 mg/kg	162 mg/kg	24 mgs
3	AB13	3,000 mg/kg	810 mg/kg	162 mg/kg	24 mgs
4	AB14	2,100 mg/kg	567 mg/kg	113.4 mg/kg	17 mgs
5	AB15	3,000 mg/kg	810 mg/kg	162 mg/kg	24 mgs

Name of the molecule: code name given for the molecule.

LD_50_ oral: Lethal dose 50: where 50% of the animals in the group have died.

LD_50_ip: LD50 oral*0.27.

ED_50_: Effective dose 50: where 50% of the animals produced results, which is one-fifth of LD_50_ ip.

IP/ip = Intraperitoneal route.

Dose of diclofenac as the standard agent is 7.5 mg/kg.

**FIGURE 8:**
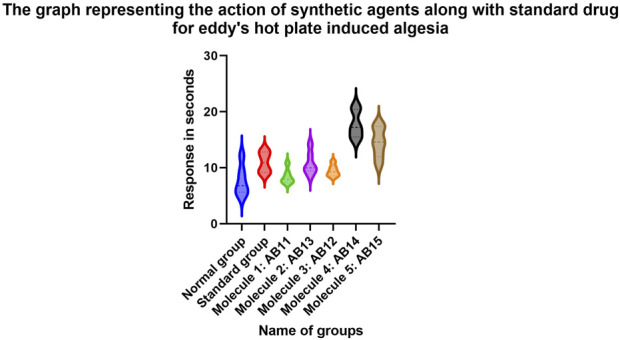
Graphs representing the results of the experiment based on Eddy’s hot plate method.

**FIGURE 9:**
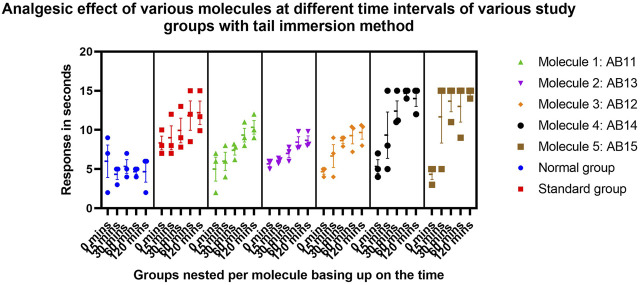
Graphs representing the results of the experiment based on the tail immersion method.

It is evident that the standard drug, i.e., diclofenac, is highly effective as an analgesic agent in controlling and reducing algesia against both the described models. Molecules AB11, AB13, AB12, AB14, and AB15 are significantly equal in potency with the standard drug in reducing algesia episodes. However, molecules AB14 and AB15 are exceptional against algesia induced via Eddy’s hot plate method and produced results similar to those of the standard; and, in some cases, they were found to produce better results than the standard drugs. In case of the tail immersion method, all the molecules produced better results than the control group of animals, and molecules AB14 and AB15 produced similar results to that of the standard. Hence, through this study, it can be concluded that the synthetic molecules are better analgesic agents (Table 2; Tables 7–10) (Figures 1, 8, 9).

**TABLE 8:** Effect of synthetic agents on tail-immersion-induced algesia.

Treatment	Response in seconds mean ± SEM (secs) 0 min	Response in seconds mean ± SEM (secs) 15 min	Response in seconds mean ± SEM (secs) 30 min	Response in seconds mean ± SEM (secs) 60 min	Response in seconds mean ± SEM (secs) 120 min
Control group (distilled water)	6 ± 2.082	4.333 ± 0.6667	5.333 ± 0.8819	4.667 ± 0.3333	4.667 ± 1.333
Standard group (diclofenac)	8.333 ± 0.8819	9 ± 1.528	9.933 ± 1.572	11.83 ± 1.878	12.2 ± 1.493
AB11	5 ± 1.528	6 ± 1.155	7.4 ± 0.611	9.333 ± 0.8819	10.33 ± 0.8819
AB12	4.667 ± 0.3333	6.667 ± 1.453	8.633 ± 0.3667	9.267 ± 1.035	9.667 ± 0.8353
AB13	5.667 ± 0.3333	6.1 ± 0.2082	7 ± 0.5292	8.433 ± 0.6839	8.667 ± 0.5696
AB14	6.000 ± 2.082	20.22 ± 1.421	21.33 ± 1.062	20.03 ± 1.737	21.50 ± 1.384
AB15	4.333 ± 0.6667	11.67 ± 3.333	13.67 ± 1.333	13 ± 2	14.67 ± 0.3333

**TABLE 9:** Effect of synthetic agents on Eddy’s hot-plate- and tail-immersion-induced algesia.

Treatment	Eddy’s hot plate mean ± SEM (secs)	Tail immersion mean ± SEM (secs)
Control group (distilled water)	7.647 ± 1.111##	5 ± 0.3055####
Standard group (diclofenac)	10.96 ± 0.7475**	10.26 ± 1.455****
AB11	8.423 ± 0.5414###	7.613 ± 0.8873**##
AB12	9.467 ± 0.4171	7.78 ± 0.7855**#
AB13	10.77 ± 0.8382**	7.173 ± 0.1798*##
AB14	17.72 ± 0.9941****####	11.15 ± 1.131****
AB15	14.52 ± 1.103****##	11.47 ± 1.338****

Two-way ANOVA, multiple comparison: Dunnett’s test.

* ≤0.05 is considered significant, ** ≤0.01 is considered highly significant, *** ≤0.01 is considered extremely significant, and **** ≤0.01 is considered very extremely significant in comparison against the control group.

# ≤ 0.05 is considered significant, ## ≤ 0.01 is considered highly significant, ### ≤ 0.01 is considered extremely significant, and #### ≤ 0.01 is considered very extremely significant in comparison against the standard group.

**TABLE 10:** Effect of synthetic agents on Eddy’s hot-plate-induced algesia.

Treatment	Response in seconds mean ± SEM (secs) 0 min	Response in seconds mean ± SEM (secs) 15 min	Response in seconds mean ± SEM (secs) 30 min	Response in seconds mean ± SEM (secs) 60 min	Response in seconds mean ± SEM (secs) 120 min
Control group (distilled water)	7.900 ± 1.644	8.017 ± 1.581	8.467 ± 1.455	6.600 ± 0.9096	7.250 ± 1.154
Standard group (Diclofenac)	8.200 ± 0.8767	9.800 ± 0.8112	10.45 ± 0.9959	12.85 ± 1.107	13.50 ± 0.8287
AB11	6.733 ± 0.3303	7.317 ± 0.3027	7.750 ± 0.8241	9.183 ± 1.018	11.13 ± 0.8437
AB12	5.167 ± 0.6009	8.883 ± 0.9948	11.65 ± 0.5655	10.43 ± 0.8285	11.20 ± 1.150
AB13	6.033 ± 0.4897	10.50 ± 0.8528	10.63 ± 0.6805	12.95 ± 1.997	13.75 ± 1.963
AB14	5.500 ± 0.4282	20.22 ± 1.421	21.33 ± 1.062	20.03 ± 1.737	21.50 ± 1.384
AB15	6.500 ± 0.7638	14.83 ± 1.014	17.23 ± 1.396	16.12 ± 2.835	17.90 ± 2.445

**FIGURE 10:**
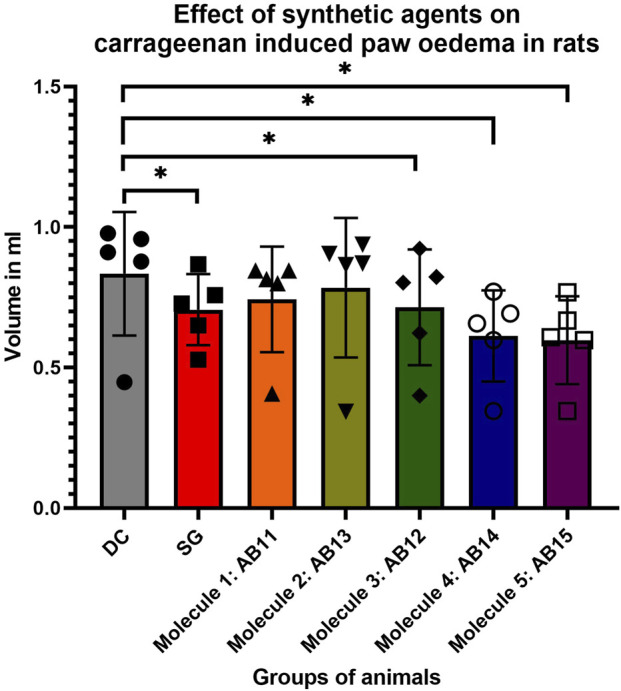
Effect of synthetic agents on carrageenan-induced paw edema in rats.

It is evident that the standard drug diclofenac is highly effective as an anti-inflammatory agent in controlling carrageenan-induced swelling in the given model. Molecules AB12, AB14, and AB15 have shown significant potency in comparison with the standard drug in reducing the rat paw swelling over time. However, molecules AB14 and AB15 are exceptional against carrageenan-induced paw edema via the inflammation method and has shown results similar to that of the standard agent; they were found to produce better and equal results to that of the standard drug. Hence, it is concluded that the synthetic molecules are better anti-inflammatory agents (Figure 10; Table 11).

**TABLE 11:** Effect of synthetic agents on carrageenan-induced paw edema in rats (mean ± SD).

Treatment	0 min	45 min	90 min	135 min	180 min
Control group (distilled water)	0.4475 ± 0.1159	0.8775 ± 0.05679	0.9100 ± 0.05477	0.9775 ± 0.08302	0.9575 ± 0.04717
Standard group (diclofenac)	0.5275 ± 0.1672	0.8675 ± 0.05315	0.7275 ± 0.05560	0.7575 ± 0.09215	0.6500 ± 0.05888**
AB11	0.4075 ± 0.09430	0.8450 ± 0.03697	0.8450 ± 0.04041	0.8000 ± 0.06683	0.8150 ± 0.04509
AB12	0.4000 ± 0.1074	0.9225 ± 0.04193	0.8025 ± 0.1284	0.6230 ± 0.3675*	0.8225 ± 0.04992*
AB13	0.3425 ± 0.05377	0.9375 ± 0.1871	0.9050 ± 0.08737#	0.8700 ± 0.05657	0.8650 ± 0.07550
AB14	0.3450 ± 0.05323	0.7700 ± 0.04899	0.6925 ± 0.1164	0.6575 ± 0.1323	0.5975 ± 0.04573*
AB15	0.3450 ± 0.05196	0.7675 ± 0.05315	0.6675 ± 0.05315*	0.5975 ± 0.009574*	0.6075 ± 0.05909*

The obtained raw data were tested through one-way analysis of variance (one-way ANOVA), selecting Tukey’s test as post-test against the base and standard treated groups to obtain the significance via GraphPad Prism.

* is used to express significance against the control group.

# is used to express significance against the standard group.

*≤ 0.05 is considered significant.

** ≤0.01 is considered highly significant.

*** ≤0.001 is considered extremely significant.

## 4 Discussion

The five Schiff bases obtained yielded a percentage yield between 79% and 84%, indicating the efficient and reproducible synthetic procedures employed. The synthesized Schiff base derivatives exhibited notable biological activity, which can be attributed to the inherent properties of the Schiff base scaffold, contributing to their improved anti-inflammatory activity. The current synthetic agents offer better yield and reproducibility with structural stability, making them perfect for pharmacological evaluation. This suggests that the molecules used here may enhance the interactions with the pharmacological targets such as cyclo-oxygenase 2 enzymes, thereby improving the pharmacological efficacy and introducing a novel lead into the anti-inflammatory class of agents (Rashmi et al., 2021).

### 4.1 Molecular docking and molecular dynamic simulation

The molecular docking results with lowest binding energies of −10 kcal/mol were seen with ligand AB 15; however, the ligand with −9.6 kcal/mol indicating a stable bound ligand, AB 12, with protein COX-2 (Kataria et al., 2025) was selected because of stable hydrogen bonds with the later ligand than the prior one. The best pose of the docked complex with RMSD = 0 was selected for the 50-ns MD simulation via GROMACS, which yielded a clear topological, index, and energy, volume, and potential minimized files for performing a successful run of simulation. The results yielded a thermodynamically stable and folded complex with stable bonds. The RMSD with stable plateau, RMSF with least fluctuations, stable hydrogen bonds ranging between 0 and 3, lowest potential energy of −685,000 kj/mol, and stable radius of gyration of 2.5 nm for the protein and 0.4 nm for the ligand showed that the system reached equilibrium well within the given time with stable structural integrity, and the study expresses that the results are within the limits of the range for a stable outcome (Fouda et al., 2025; Abdelsadek et al., 2020; Germoush et al., 2025). A study targeting the same protein COX-2 with a different crystal structure simulated with a ligand also showed a stable but lower value compared to the current study (Selvaraj et al., 2020). The docking and simulating poses observed in the current study were also stable when compared with those of a similar published study (Selvaraj et al., 2020). The current study consists of five novel synthetic ligands, of which AB 15 and AB 12 are exceptional according to the docking and simulation results, and these ligands were pre-clinically tested using rats to justify the outcomes related to anti-inflammatory and analgesic actions.

The total decomposition analysis using MM–PBSA and MM–GBSA of the complexes showed that for AB 12, the ΔG_bind value, which is slightly positive, shows that binding does not occur by itself in the simulated conditions, which can be explained by the results of energy decomposition. The analysis shows a classic thermodynamic profile, with strong, specific interactions in the bound state being strongly countered by the high energy cost of the dehydrating polar and charged groups. For instance, residues such as ARG44, LYS137, and ARG469 show that strong electrostatic interactions can be hidden in the net energy due to high de-solvation and their charged side chains. The case of GLU465 is especially useful because its unfavorable electrostatic and polar solvation energies in the complex suggest that it is an important residue that is stabilized in the unbound (solvated) state and must undergo extensive de-solvation and conformational rearrangement while binding. This suggests that the binding interface has a lot of polarity, and the benefits of certain hydrogen bonds and salt bridges are mostly used to eliminate water molecules from the interacting surfaces. The large standard deviations for some energy components, especially the electrostatic and polar solvation terms, show how dynamic these interactions are. This suggests that entropy or certain conformational states that were not fully captured in this calculation may be important for obtaining good binding in a physiological context. In addition, in the case of the standard molecule diclofenac, identifying essential target residues such as HIS:39, LEU:152, and GLN:461 gives a better understanding of the manner in which the ligand (diclofenac) binds to the protein (COX-2). The ligand’s significant contribution illustrates its ideal placement within the binding pocket. The classic energetic signature for LIG:584, which shows a very favorable gas-phase electrostatic energy and a substantial polar solvation charge, indicates charged or highly polar interactions. This suggests that the binding mechanism comprises durable, specific interactions such as hydrogen bonds or salt bridges, which are partially dissolved during complex formation. The significant van der Waals interactions from both the ligand (diclofenac) and hydrophobic residues of proteins, such as LEU:152 and VAL:46, demonstrate the significance of the complementary form and hydrophobic packing for retaining the stability of the complex. These results demonstrate which residues are important for binding and explain how they interact with each other. These data will be helpful for future structure-based drug design with the goal of improving affinity or specificity.

### 4.2 Preclinical study discussion

The current study was designed to evaluate the acute oral toxicity and the analgesic and anti-inflammatory activity of five synthetic compounds, AB11, AB13, AB12, AB14, and AB15, in murine models. The acute oral toxicity studies revealed that the compounds have LD_50_ values ranging from 2,100 to 3,000 mg/kg, which indicates moderate toxicity (Tzvetkov et al., 2014). The oral toxicity values were converted to intraperitoneal doses by multiplying a correction factor of 0.27 for safer and easier administration and the subsequent selection of one-fifth of the IP LD_50_ as the test dose (ED_50_) for the sake of efficacy studies, for which the final calculated test doses were identified between 17 mg/kg and 24 mg/kg, for assessing the analgesic and anti-inflammatory effects while minimizing adverse effects.

In case of analgesic evaluation through Eddy’s hot plate and tail immersion methods, the compounds showed varied degrees of efficacy (Khalturina et al., 2010; Chahal et al., 2023) in comparison to the standard drug diclofenac, which exhibited significant analgesic and anti-inflammatory activity, as expected. Among the test compounds, AB14 and AB15 showed exceptional activity, which was comparable to that of diclofenac, especially in Eddy’s hot plate method, and both the compounds showed significant results (p ≤ 0.01) and were extremely significant in pain response latency (p ≤ 0.0001), suggesting strong central analgesic behavior. Similarly in the tail immersion test, AB14 and AB15 exhibited potent analgesic effects, which confirms their potency as effective pain-relieving agents. The three remaining compounds, AB11, AB13, and AB12, also proved to be significant analgesic agents but to a lesser extent compared to AB14 and AB15.

The anti-inflammatory potential was assessed using a carrageenan-induced paw edema rat model. The standard drug diclofenac significantly reduced the inflammation over time, serving as the positive control. The two compounds AB14 and AB15 contributed to significant enhancement in the management of inflammation, i.e., p ≤ 0.05, and highly significant (p ≤ 0.01) reduction in paw edema, which can be comparable to that of diclofenac. The compound AB 12 also showed better anti-inflammatory effects, but it was less in comparison to those of AB14 and AB15. These findings suggest that the molecules AB 14 and AB15 are excellent analgesic and anti-inflammatory agents and can be considered strong candidates for future pharmacological developments. Thus, the incorporation of the 1-aminoindazole moiety was found to enhance COX-2 selectivity, and the pharmacological data obtained in this study support the initial hypothesis by demonstrating improved activity compared with the parent scaffolds.

## 5 Conclusion

The Schiff bases are synthesized with the highest yield, and better structural stability suggests enhanced pharmacological activity toward the targets of interest, i.e., COX-2 enzymes. The pharmacological evaluation of the compounds demonstrated that the synthetic compounds, i.e., AB14 and AB15, exhibited potent analgesic and anti-inflammatory effects, which is comparable to that of diclofenac in certain models, as substantiated by the obtained murine study data. Their ability to reduce pain and inflammation classifies them as potent novel therapeutic agents for further screening. Future studies must include receptor-binding capability analysis; chronic toxicity evaluation can be performed to elucidate their mechanism of action and expand their safety profile before clinical translation. These leads would provide a better paradigm for assisting in future synthetic analgesic and anti-inflammatory drug discoveries from the research perspective.

## 6 Limitations and future directions

The optimization of ligands in the current research was conducted using ChemDraw 3D, which offered initial stabilization but does not possess the rigor of force-field-based methodologies such as MMFF94. This limitation could affect how well docking works; therefore, future work should use more advanced optimization methods and longer simulations to make the results more reliable and provide better insights into SAR.

**FIGURE 11:**
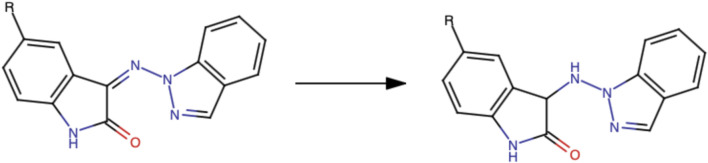
Scheme employed in the synthesis of the active molecules.

This synthetic part of the research work will be extended to include reduction of the prepared hydrazones (10) to the corresponding hydrazine derivatives (16) (Figure 11) and examine their anti-inflammatory and analgesic activities in comparison with those of hydrazones and determine if they have COX-1 or COX-2 anti-inflammatory activity. The current study evaluates their percentage yield, structural stability, binding affinities, and their potency through preclinical evaluation. However, toxicity profiling of the compounds needs to be documented with in-depth analysis utilizing sub-acute toxicity studies, which would prove the potency of the molecules as potent analgesics and anti-inflammatory agents; as the route-to-route extrapolation, the intraperitoneal LD_50_ was estimated from the oral LD_50_ using a standard conversion factor (LD_50_ = LD_50_ × 0.27), as described in toxicological scaling as cited above in the *Methods* section, which also needs further in-depth study. The Schiff base derivatives demonstrated significant anti-inflammatory and analgesic activity *in vivo*, with docking studies suggesting COX-2 as a potential target. However, confirmation of this mechanism requires *in vitro* biochemical validation. Future work will, therefore, focus on COX-2 inhibition assays to substantiate the docking predictions and clarify the compounds’ mechanism of action.

## Data Availability

The related data of molecular docking and dynamic simulation is available at https://figshare.com/s/51c27873819b6f4b8064 (doi: 10.6084/m9.figshare.30675641). Further inquiries can be directed to the corresponding author(s).
