# Correction: Synthesis, characterization, and *in silico* and *in vivo* profiling of selective cyclo-oxygenase-2 inhibitors of indazole–indolinone derivatives with anti-inflammatory and analgesic potency

**DOI:** 10.3389/fphar.2026.1880153

**Published:** 2026-06-08

**Authors:** Sultan Ibrahim Alkubaysi, Mohammad Jaffar Sadiq Mantargi, Faisal Ateeq Almalki, Alaa Mohammad Alqahtani, Alaa Omar Baryyan, Saeed Mohammed Tayeb, Hussni Ahmad Muathen

**Affiliations:** 1 Department of Pharmaceutical Sciences, College of Pharmacy, Umm Al-Qura University, Makkah, Saudi Arabia; 2 Department of Pharmaceutical Sciences, Pharmacy Program, Batterjee Medical College, Jeddah, Saudi Arabia

**Keywords:** analgesic, anti-inflammatory, drug development, *in silico*, molecular docking, molecular dynamic simulations, preclinical study

Affiliation “Department of Pharmaceutical Sciences, College of Pharmacy, Umm Al-Qura University, Makkah, Saudi Arabia” was erroneously given as “Pharmaceutical Chemistry, Pharmacy College, Umm Al-Qura University, Makkah, Saudi Arabia”.

Additionally, the author’s name “Saeed Mohammed Tayeb” was erroneously given as “Saeed Mohammad Tayeb”.

The original version of this article has been updated.

