# Supplementary material for: Synthesis, characterization, and in silico and in vivo profiling of selective cyclo-oxygenase-2 inhibitors of indazole–indolinone derivatives with anti-inflammatory and analgesic potency
Source: Front Pharmacol. 2026 Jan 30;16:1723200. doi: 10.3389/fphar.2025.1723200 (PMC12902688; doi:10.3389/fphar.2025.1723200)
Supplement: Supplementary file 1 [file Supplementaryfile1.pdf]

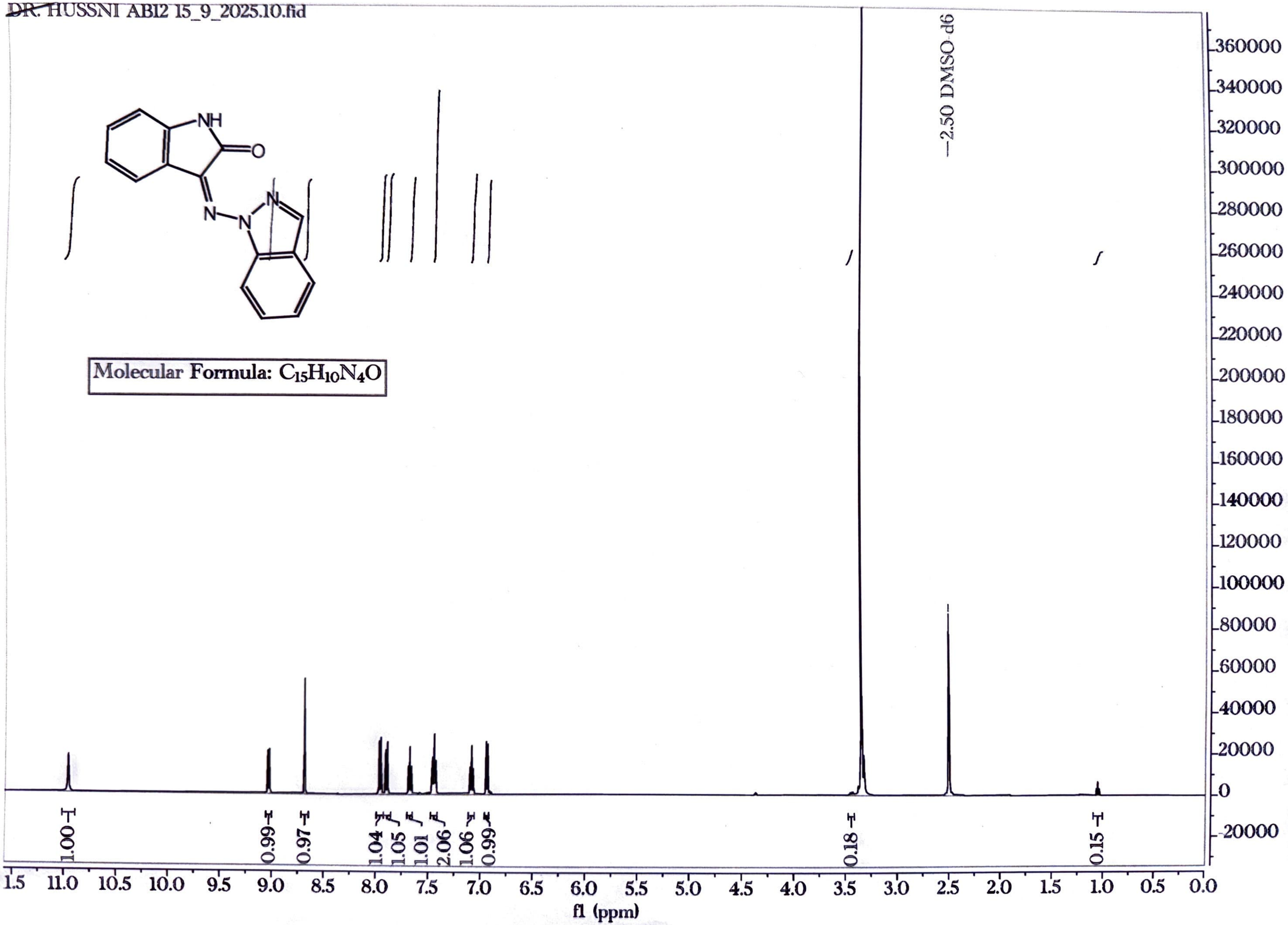

DR. HUSSNI AB12 15_9_2025.10.fid
NH
O
N
N
N
Molecular Formula: C15H10N4O
—2.50 DMSO-d6
1.00
0.99
0.97
1.04
1.05
1.01
2.06
1.06
0.99
0.18
0.15
360000
340000
320000
300000
280000
260000
240000
220000
200000
180000
160000
140000
120000
100000
80000
60000
40000
20000
0
-20000
11.5 11.0 10.5 10.0 9.5 9.0 8.5 8.0 7.5 7.0 6.5 6.0 5.5 5.0 4.5 4.0 3.5 3.0 2.5 2.0 1.5 1.0 0.5 0.0
f1 (ppm)

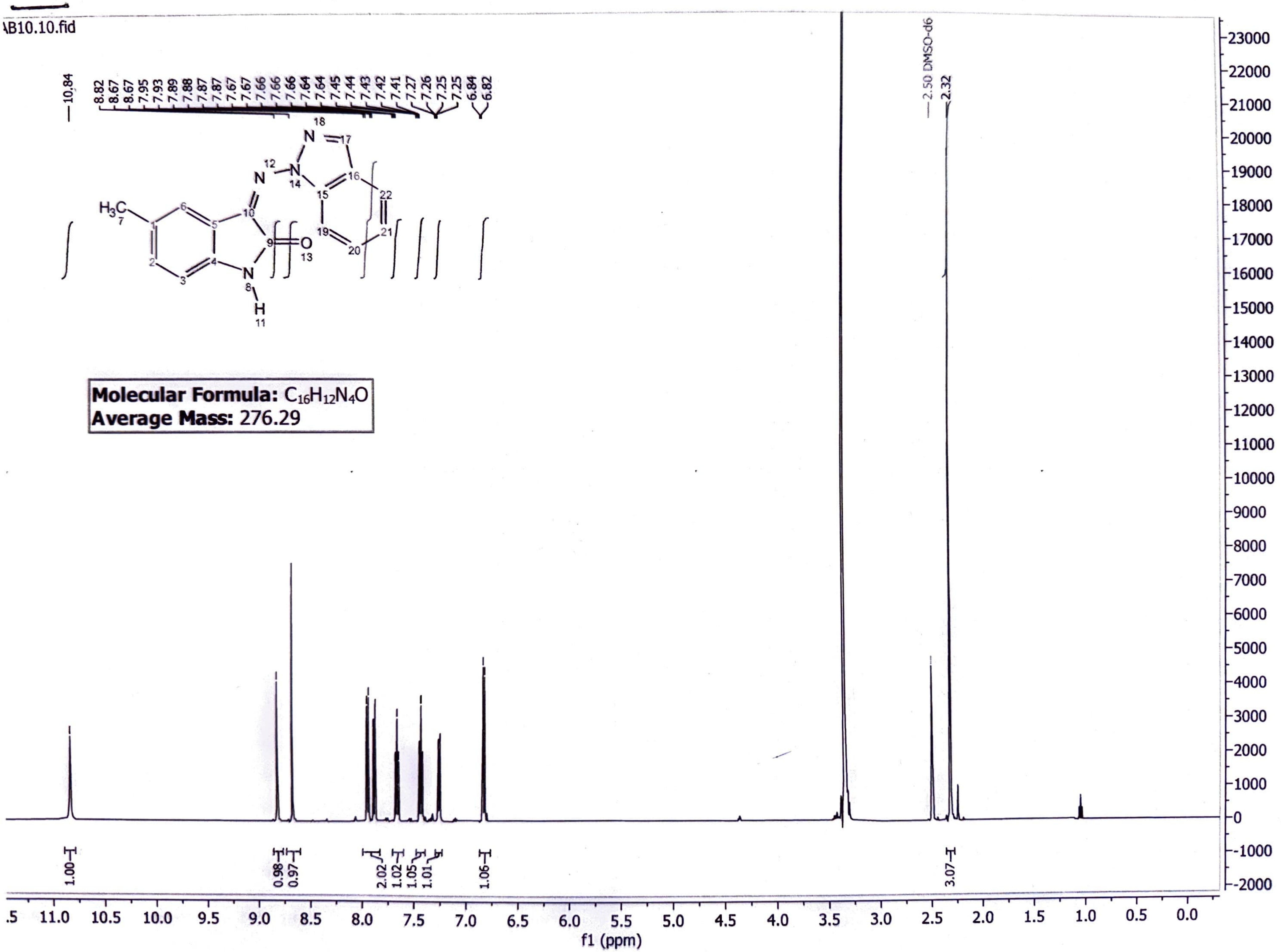

AB10.10.fid
10.84
8.82
8.67
8.67
7.95
7.93
7.89
7.88
7.87
7.87
7.67
7.67
7.66
7.66
7.66
7.64
7.64
7.45
7.44
7.43
7.42
7.41
7.27
7.26
7.25
7.25
6.84
6.82
2.50 DMSO-d6
2.32
H3C
Molecular Formula: C16H12N4O
Average Mass: 276.29
1.00
0.98
0.97
2.02
1.02
1.05
1.01
1.06
3.07
f1 (ppm)

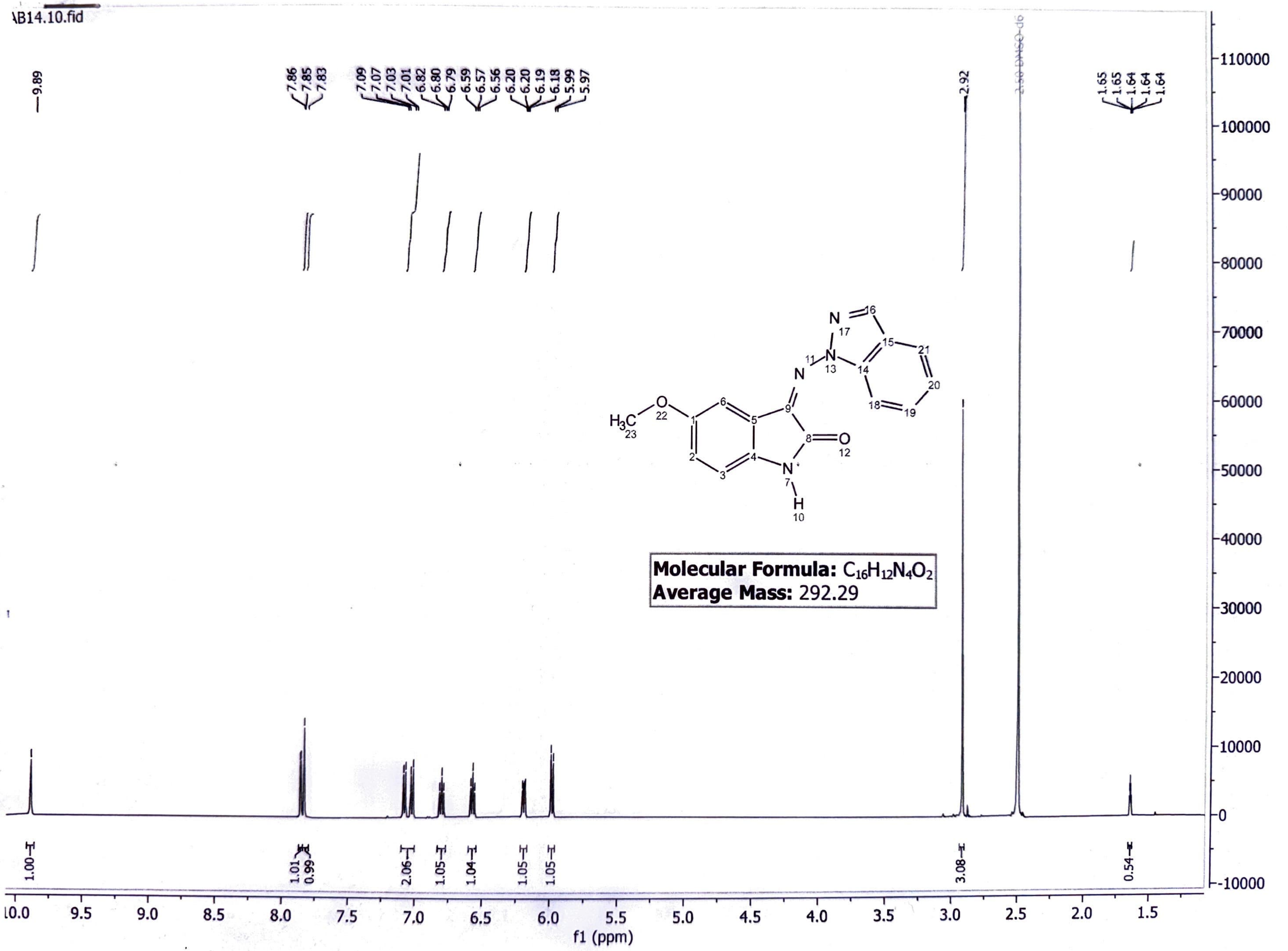
B14.10.fid
9.89
7.86
7.85
7.83
7.09
7.07
7.03
7.01
6.82
6.80
6.79
6.59
6.57
6.56
6.20
6.20
6.19
6.18
5.99
5.97
2.92
2.50 DMSO-d6
1.65
1.65
1.64
1.64
1.64
1.00
1.01
0.99
2.06
1.05
1.04
1.05
1.05
3.08
0.54
f1 (ppm)
H3C
O
N
N
N
N
H
O
Molecular Formula: C16H12N4O2
Average Mass: 292.29

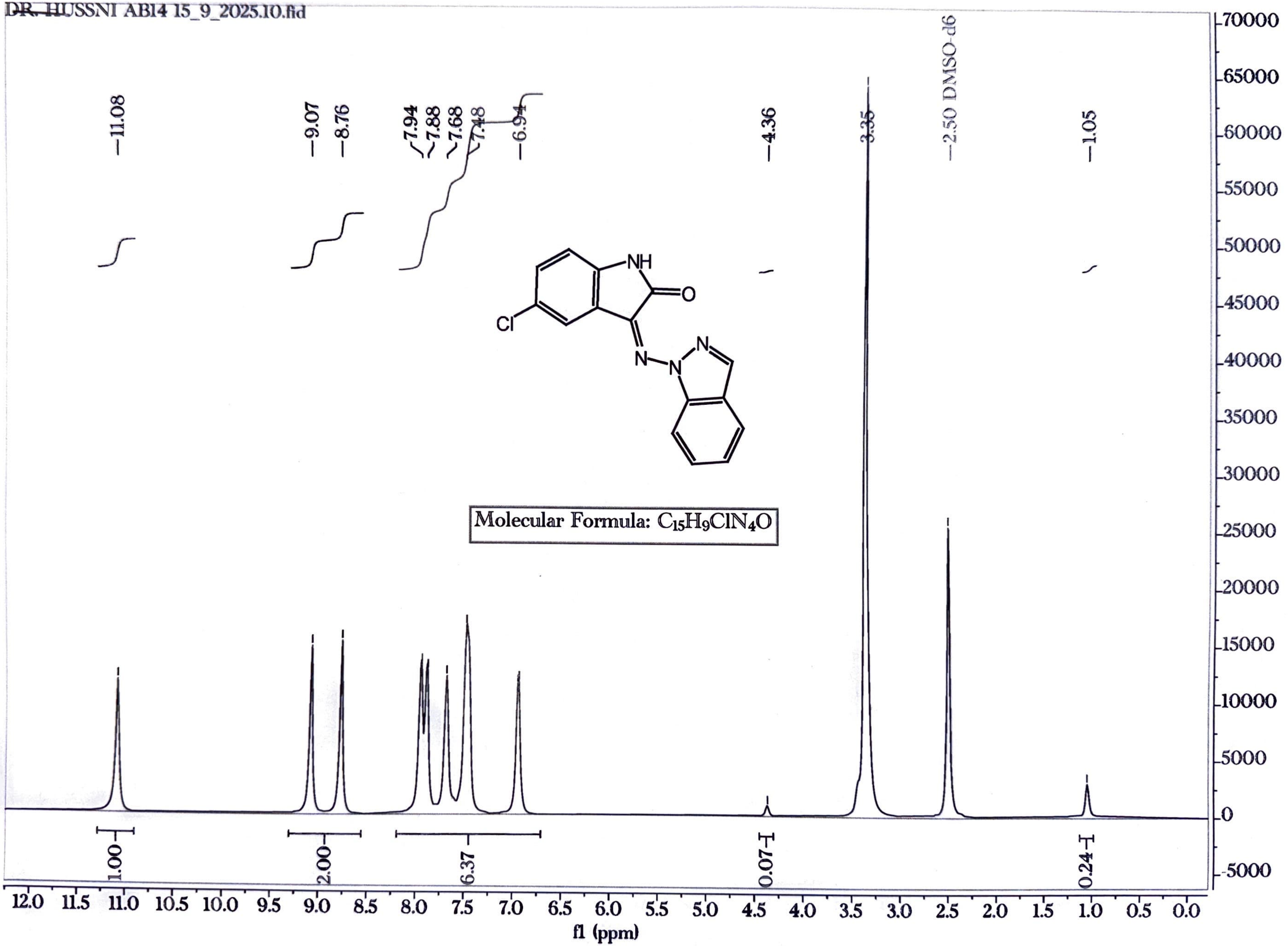
DR. HUSSNI AB14 15_9_2025.10.fid
11.08
9.07
8.76
7.94
7.88
7.68
7.48
6.94
4.36
3.35
2.50 DMSO-d6
1.05
NH
O
Cl
N
N
N
Molecular Formula: $C_{15}H_9ClN_4O$
1.00
2.00
6.37
0.07
0.24
12.0 11.5 11.0 10.5 10.0 9.5 9.0 8.5 8.0 7.5 7.0 6.5 6.0 5.5 5.0 4.5 4.0 3.5 3.0 2.5 2.0 1.5 1.0 0.5 0.0
f1 (ppm)
70000 65000 60000 55000 50000 45000 40000 35000 30000 25000 20000 15000 10000 5000 0 -5000

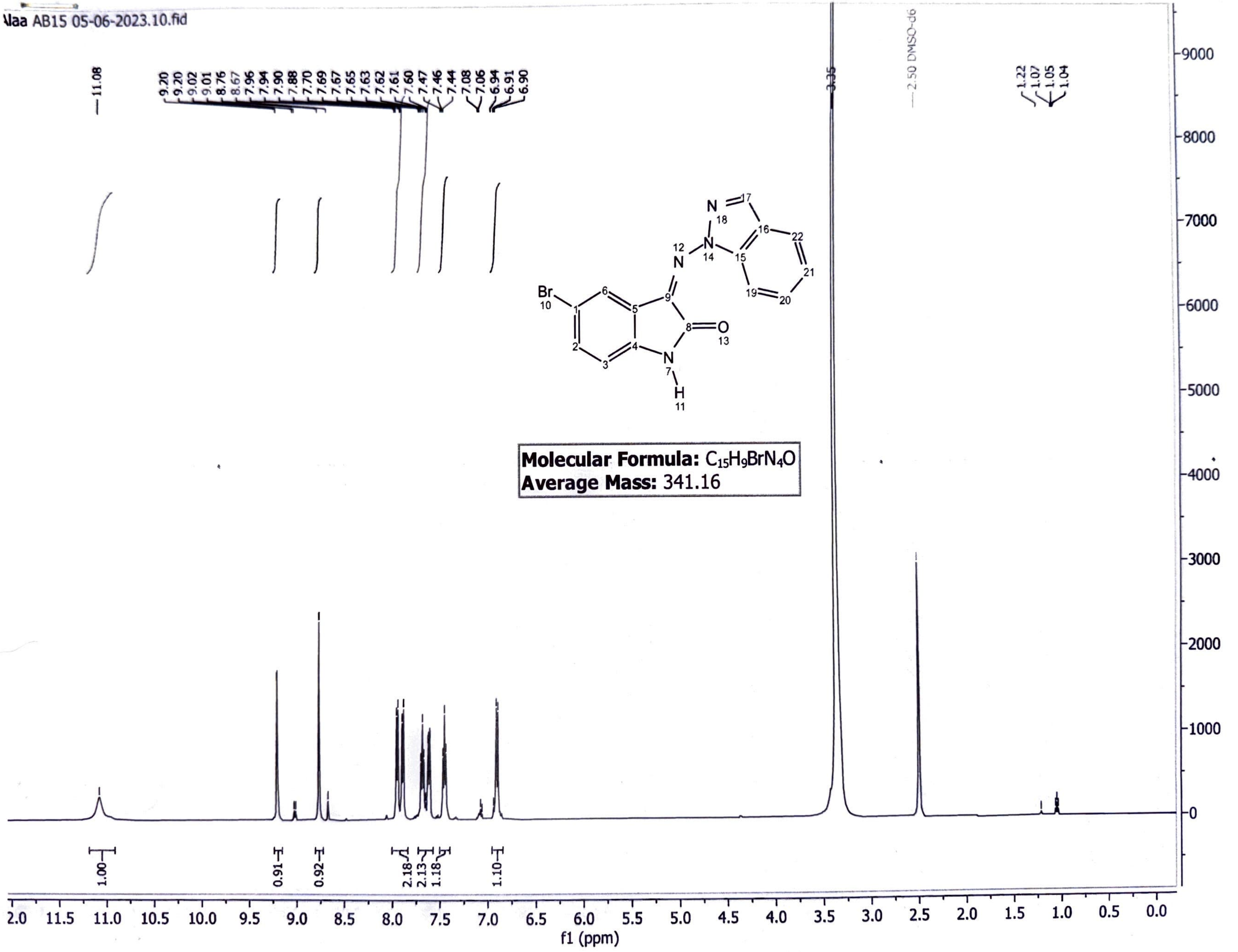

laa AB15 05-06-2023.10.fid
11.08
9.20 9.20 9.02 9.01 8.76 8.67 7.96 7.94 7.90 7.88 7.70 7.69 7.67 7.65 7.63 7.62 7.61 7.60 7.47 7.46 7.44 7.08 7.06 6.94 6.91 6.90
3.35
2.50 DMSO-d6
1.22 1.07 1.05 1.04
Br
10
N
12
14
18
N
11
13
7
H
O
Molecular Formula: $C_{15}H_9BrN_4O$
Average Mass: 341.16
1.00
0.91
0.92
2.18
2.13
1.18
1.10
9000
8000
7000
6000
5000
4000
3000
2000
1000
0
2.0 11.5 11.0 10.5 10.0 9.5 9.0 8.5 8.0 7.5 7.0 6.5 6.0 5.5 5.0 4.5 4.0 3.5 3.0 2.5 2.0 1.5 1.0 0.5 0.0
f1 (ppm)

SHIMADZU

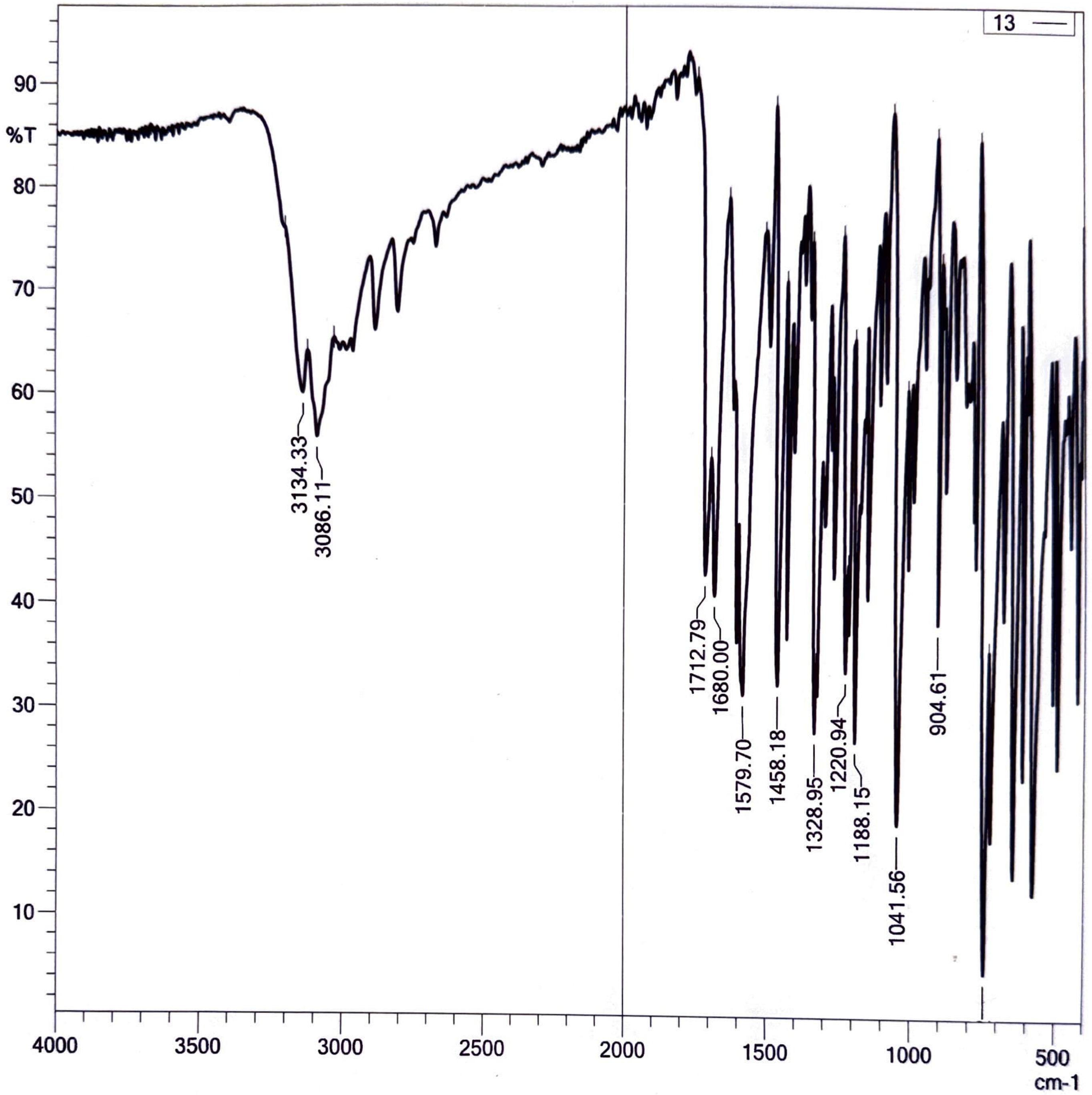

C:¥Users¥User¥Desktop¥Alaa¥Dr. Husni¥13.ispd

77%

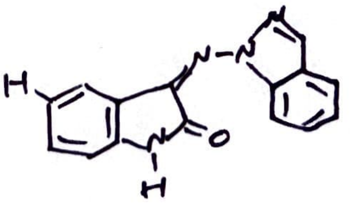

	Item	Value
2	Sample name	AB10
3	Sample ID	
4	Option	
5	Intensity Mode	%Transmittance
6	Apodization	Happ-Genzel
9	No. of Scans	20

SHIMADZU

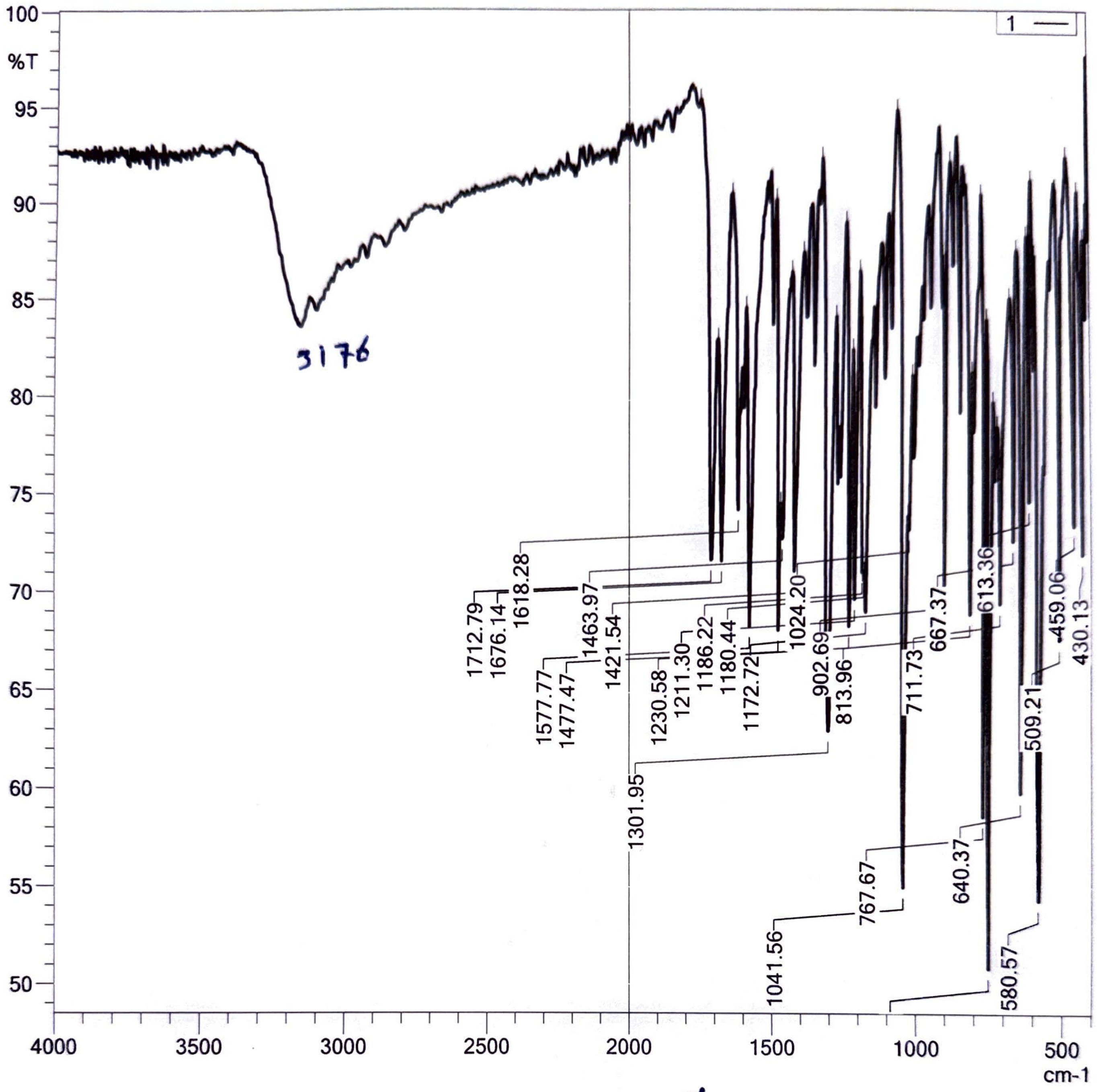

C:¥Users¥User¥Desktop¥Alaa¥Dr. Husni¥1.ispd

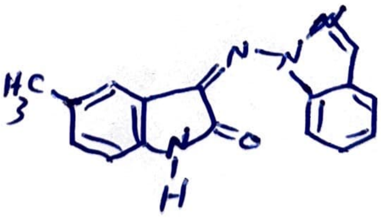

	Item	Value
2	Sample name	AB10
3	Sample ID	
4	Option	
5	Intensity Mode	%Transmittance
6	Apodization	Happ-Genzel
9	No. of Scans	20

SHIMADZU

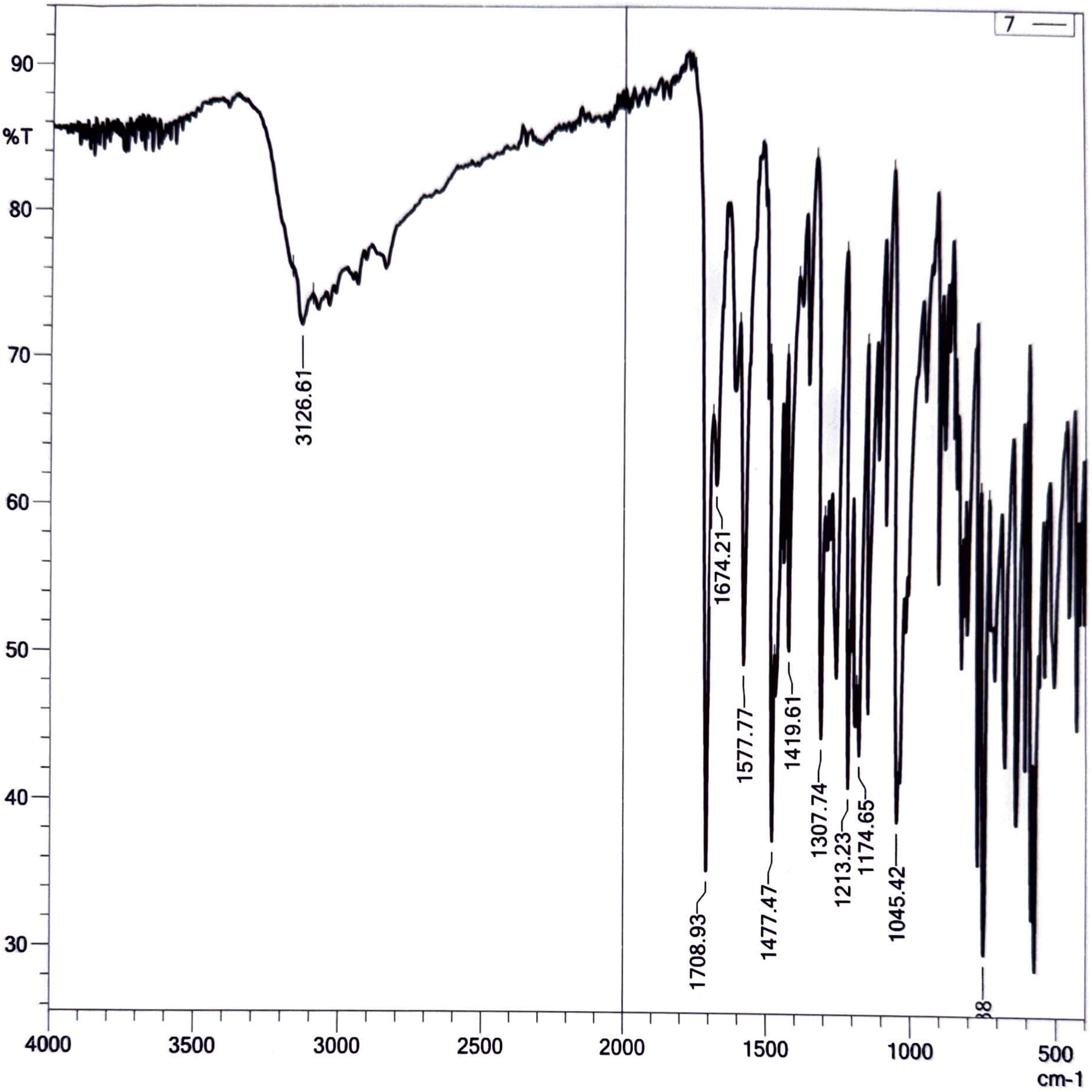

C:¥Users¥User¥Desktop¥Alaa¥Dr. Husni¥7.ispd

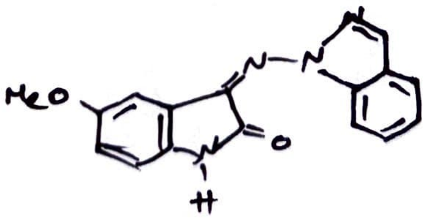

	Item	Value 0·39
2	Sample name	AB10
3	Sample ID	
4	Option	
5	Intensity Mode	%Transmittance
6	Apodization	Happ-Genzel
9	No. of Scans	20

SHIMADZU

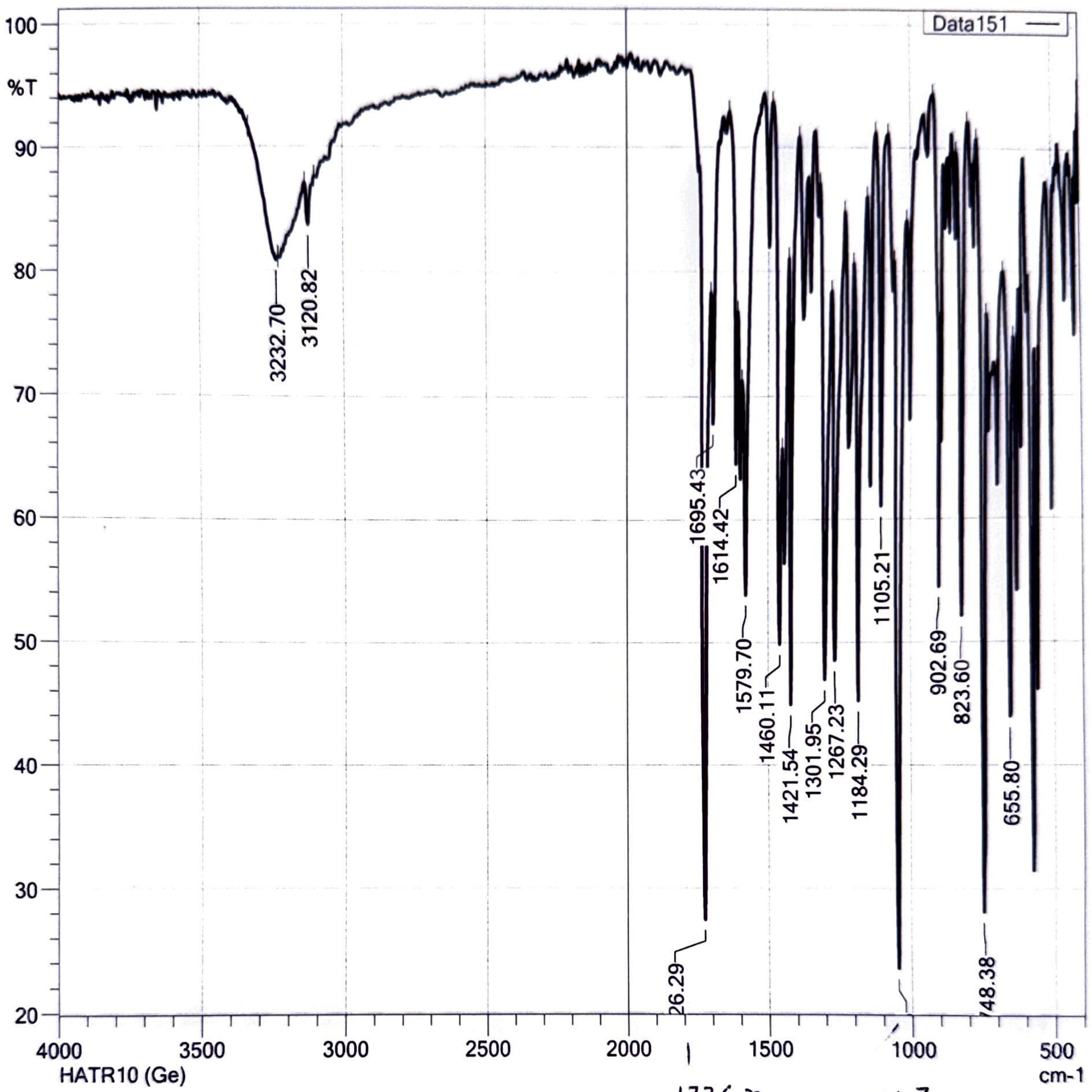

1726.29 1047 748

C:¥LabSolutions¥LabSolutionsIR¥Data¥Data151.ispd

HATR10 (Ge)

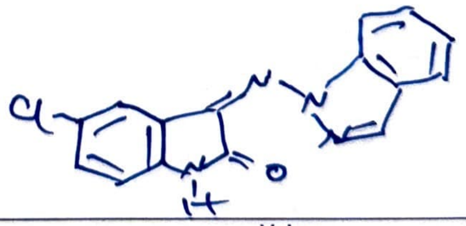

2

	Item	Value
2	Sample name	
3	Sample ID	
4	Option	
5	Intensity Mode	%Transmittance
6	Apodization	Happ-Genzel
9	No. of Scans	30

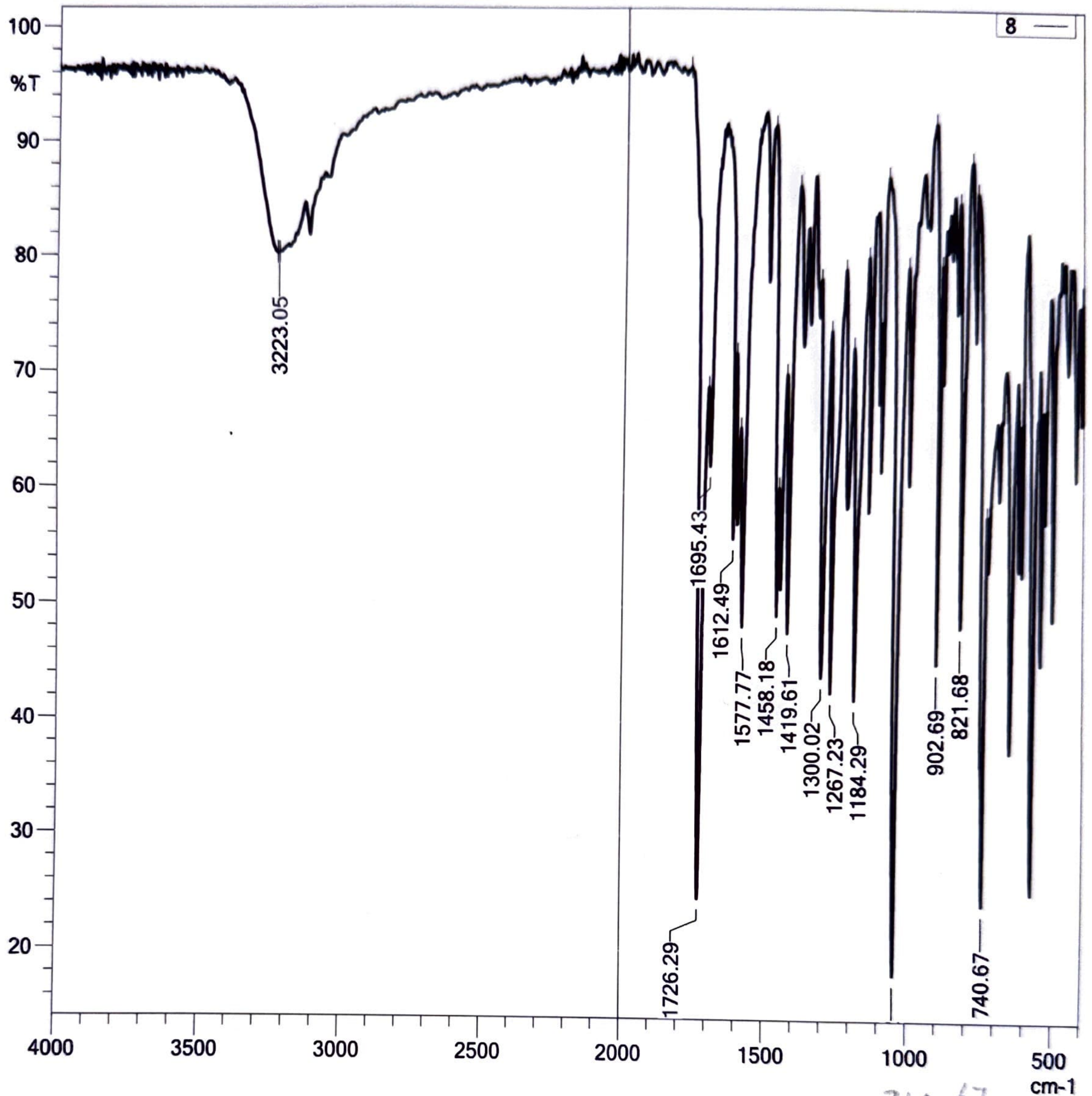

740.67

C:¥Users¥User¥Desktop¥Alaa¥Dr. Husni¥8.ispd

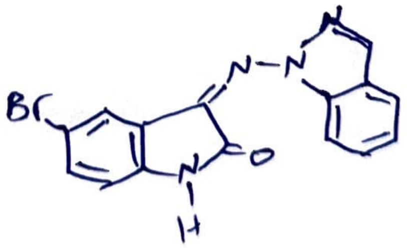

79%

	Item	Value
2	Sample name	AB10
3	Sample ID	
4	Option	
5	Intensity Mode	%Transmittance
6	Apodization	Happ-Genzel
9	No. of Scans	20
